# A nucleoskeleton network preserves genomic integrity by promoting NHEJ and restraining chromosome translocations

**DOI:** 10.1093/nar/gkaf1354

**Published:** 2025-12-08

**Authors:** Jingyan Liu, Xiuzhen Bai, Xinpeng Chen, Bohan Li, Huayu Zhao, Jiahui Wu, Yuanling Ye, Jiayi Yu, Zhenxin Yan, Rong Guo, Dongyi Xu, Wen Li

**Affiliations:** Center for Medical Epigenetics, School of Basic Medical Sciences, Chongqing Medical University, Chongqing 400016, China; State Key Laboratory of Gene Function and Modulation Research, School of Life Sciences, Peking University, Beijing 100871, China; State Key Laboratory of Gene Function and Modulation Research, School of Life Sciences, Peking University, Beijing 100871, China; Biomedical Pioneering Innovation Center (BIOPIC), Peking University, Beijing 100871, China; State Key Laboratory of Gene Function and Modulation Research, School of Life Sciences, Peking University, Beijing 100871, China; State Key Laboratory of Gene Function and Modulation Research, School of Life Sciences, Peking University, Beijing 100871, China; State Key Laboratory of Gene Function and Modulation Research, School of Life Sciences, Peking University, Beijing 100871, China; State Key Laboratory of Gene Function and Modulation Research, School of Life Sciences, Peking University, Beijing 100871, China; Center for Medical Epigenetics, School of Basic Medical Sciences, Chongqing Medical University, Chongqing 400016, China; Center for Medical Epigenetics, School of Basic Medical Sciences, Chongqing Medical University, Chongqing 400016, China; State Key Laboratory of Gene Function and Modulation Research, School of Life Sciences, Peking University, Beijing 100871, China; State Key Laboratory of Gene Function and Modulation Research, School of Life Sciences, Peking University, Beijing 100871, China; State Key Laboratory of Gene Function and Modulation Research, School of Life Sciences, Peking University, Beijing 100871, China; Center for Medical Epigenetics, School of Basic Medical Sciences, Chongqing Medical University, Chongqing 400016, China

## Abstract

Chromosomal translocation (CT) is characterized by incorrect ligation between chromosome fragments when multiple DNA double-strand breaks (DSBs) exist simultaneously. DNA repair, the three-dimensional structure of the nucleoskeleton, and the spatial and temporal movement of DSB ends contribute to CTs. Our earlier research showed that Intermediate Filament Family Orphan 1 (IFFO1) links the nucleoskeleton and non-homologous end joining (NHEJ) to prevent CTs. In this study, we identified the paralog IFFO2, which, together with IFFO1, forms a complex interaction network. We demonstrate that the C-terminus of these IFFOs binds to XRCC4 to facilitate its participation in the NHEJ process. In contrast, their N-termini oversee the building of the nucleoskeleton by connecting with each other and Lamin A/C. Interestingly, IFFO1 and IFFO2 show epistatic effects in suppressing CT by anchoring broken DNA ends and have non-epistatic roles in NHEJ-mediated DSB repair. Our results define an integrated nucleoskeleton composed of IFFO1–IFFO2–Lamin A/C, and reveal its dual functions in genome stability maintenance, the promotion of end-joining, and the suppression of CT.

## Graphical Abstract

**Graphical Abstract:**
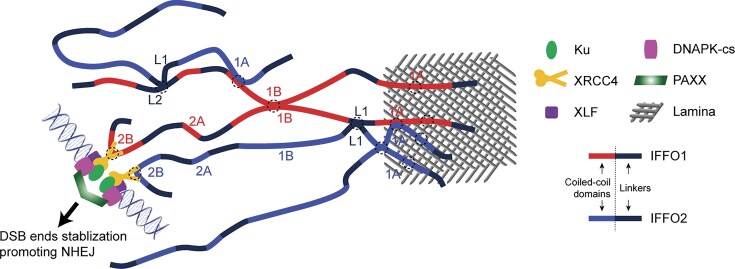

## Introduction

Intra- or interchromosomal translocations (CTs) are the result of incorrect ligation of chromosomal fragments to the same or different chromosomes, respectively [1]. The most well-known translocation is t(9;22)(q34;q11), which results in the expression of a BCR/ABL fusion protein and occurs in >90% of chronic myeloid leukemia cases [2, 3]. Parallel translocation between a strong promoter or enhancer and an oncogene may introduce new regulatory units. For example, in Burkitt lymphoma, the t(8;14)(q24;q32) translocation activates the C-MYC oncogene [2, 4]. CT is clinically associated with cancers of mesenchymal origin [5, 6], solid tumors [7, 8], and hematological malignancies [7, 8]. This process is primarily caused by the misligation of two double-strand breaks (DSBs) that can occur when cells encounter exogenous threats or endogenous physiological events [9]. Canonical non-homologous end joining (c-NHEJ) and homologous recombination (HR) are the major pathways to repair DSBs [10]. The NHEJ pathway carries out repairs throughout the cell cycle by utilizing the sensors KU70/KU80, the effector DNA-PKcs, the ligation complex ligase IV, XRCC4, XLF, and PAXX, and other end-processing factors such as Artemis and APLF [11, 12]. In the absence of c-NHEJ factors such as Ku and XLF, the ends of DNA breaks are not stabilized [13–15], and other error-prone repair pathways become prominent, such as alternative end joining (a-EJ) [16], increasing the frequency of CTs [17, 18]. However, the mechanisms driving CTs need to be broadly elucidated.

The nucleoskeleton proteins lamins form a network structure known as the “lamina” under the inner membrane of metazoan nuclei [19, 20] to organize the advanced 3D structure of the genome and regulate chromatin and nucleic acid metabolism [21]. Recent research has shown that Lamin A/C promotes DNA damage repair by maintaining the expression of 53BP1, BRCA1, and RAD51 [22, 23]. In addition, Lamin A/C maintains the stability of DNA damage sites by binding to phosphorylated H2AX (γH2AX), a marker signifying DSBs [24], and suppressing chromatin dynamics to immobilize broken DNA ends [25, 26]. Previously, we reported that another nucleoskeleton protein, Intermediate Filament Family Orphan 1 (IFFO1), maintains the stability of damaged ends by connecting to the lamina, which inhibits CT [27]. These studies suggest a link between nucleoskeleton proteins and CT, although the precise mechanism remains unclear.

Here, we identified the nucleoskeleton protein IFFO2 using immunoprecipitation (IP) and mass spectrometry (MS), built an interaction network with the previously studied IFFO1, and revealed the various functional epistatic interactions of these proteins in promoting NHEJ repair and inhibiting CT. After a DSB occurs, IFFOs interact with the NHEJ core factor XRCC4 through their C-terminal region, possibly ensuring the synapsis of the two recently broken DNA ends. In this process, IFFO1, IFFO2, and XLF play redundant roles, meaning that they are functionally complementary to each other to ensure the success of DNA repair. Throughout the nucleus, the N-termini of IFFOs interact with Lamin A/C to form the nucleoskeletal structure. This architecture serves to confine DSBs to specific spatial regions, thereby limiting their mobility. If either IFFO1, IFFO2, or Lamin A/C is missing, the integrity of the nucleoskeletal structure is disrupted, leading to the formation of a CT when DSBs from different chromosome segments meet. The functions of IFFO1, IFFO2, and Lamin A/C are equally important during this process. Our study provides information on the differentiation of the molecular mechanisms of the nucleoskeleton in different biological processes, which will help us to understand the causes of chromosomal abnormalities in diseases and provide novel ideas and methods for future medical research and treatments.

## Materials and methods

### Antibodies and reagents

The antibodies used in this study are as follows: anti-DNA-PKcs [NeoMarkers Fremont, catalog no. 423 × 812, 1:1000 for western blotting (WB)], anti-FLAG (MBL, catalog no. M185-3L, 1:2000 for WB), anti-β-actin (MBL, catalog no. M177-3, 1:5000 for WB), anti-β-tubulin (Abcam, catalog no. Ab6046, 1:2000 for WB), anti-γH2AX (Millipore, catalog no. 05-636, 1:5000 for WB), anti-Histone H3 (Novus Biologicals, catalog no. NB500-171, 1:1000 for WB), anti-IFFO1 (Abcam, catalog no. ab242130, 1:1000 for WB), anti-IFFO2 (Abcam, catalog no. ab242131, 1:1000 for WB), anti-Ku80 (Santa Cruz Biotechnology, catalog no. sc-56132, 1:250 for WB), anti-Lamin A/C (Proteintech, catalog no. 10298-1-AP, 1:1000 for WB), anti-LaminB1 (Santa Cruz Biotechnology, catalog no. sc-30264, 1:1000 for WB), anti-ligase4 (Proteintech, catalog no. 12695-I-AP, 1:1000 for WB), anti-PAXX (Abcam, catalog no. ab126353, 1:200 for WB), anti-XLF (Bethyl, catalog no. A300-730A, 1:1000 for WB), and anti-XRCC4 (Abcam, catalog no. ab145, 1:2000 for WB). Other reagents were purchased from the indicated companies: anti-FLAG M2 affinity gel (Sigma-Aldrich, A2220, 5 ml), dextrin beads (Smart-Lifesciences, SA077010), 3× flag peptide (Beyotime, P9801, 5 mg), Opti-MEM reduced serum medium (ThermoFisher Scientific, 31985-070), Hybridoma SFM (Gibco, 12 045 084), Dulbecco’s modified Eagle’s medium (DMEM) nutrient mix F12 (Gibco, 12 400 024), methylcellulose (Sigma-Aldrich, M0387, 100 g), Giemsa Stain (Sigma-Aldrich, 48900, 100 ml), doxorubicin (Sigma-Aldrich, D9891,1g), Quick-hardening (Sigma-Aldrich, 03989; 100 ml), urea (BBI, UB0148, 500 g), and ammonium sulfate (Sigma-Aldrich, A4418, 100 g).

### Cell culture, transfection, and lentivirus packaging

HEK293 and HT1080 cells were cultured in DMEM supplemented with 10% fetal calf serum (FCS) and 1% penicillin–streptomycin at 37°C with 5% CO_2_. DT40 cells were cultured in RPMI 1640 medium supplemented with 10% FCS, 1% chicken serum, 10 mM HEPES, and 1% penicillin–streptomycin mixture at 39.5°C with 5% CO_2_. Suspended HEK293 cells were cultured in Freestyle medium (Invitrogen), with 1% FCS and 1% penicillin–streptomycin added in an incubator and shaking at 130 rpm. Transfections of plasmids were carried out with PEI (polyethyleneimine; Polysciences) for HEK293 cells, and Lipo2000 transfection reagent (Mei5 Bio, MF135-01) for HT1080 cells, or with electroporation using a Lonza Nucleofector 4D for DT40 cells. The cell lines were obtained from ATCC and are not among those listed as commonly misidentified by the International Cell Line Authentication Committee. All cell lines were subjected to mycoplasma testing twice per month and were found to be negative. The identity of the cell lines was validated by short tandem repeat (STR) profiling (ATCC) and by analysis of chromosome number in metaphase spreads. For selection, growth media containing G418 (2 mg/ml), puromycin (0.5 μg/ml), blasticidine (25 μg/ml), or histidinol (1 mg/ml) were used.

Lentiviral plasmids were co-transfected into HEK293 cells using PEI for short hairpin RNA (shRNA) packaging After 4 days, the packaged lentivirus supernatants were harvested and stored at −80°C until further use. Cells transfected with shRNA lentivirus were selected with puromycin (1 μg/ml). The shRNA sequences in this study are:

shXRCC4-1: 5′-CCGGGAGTTGGTACTGGATCACATTCTCGAGAATGTGATCCAGTACCAACTCTTTTTG-3′

shXRCC4-2: 5′-CCGGCCTAATGGTCACCACTTCTTACTCGAGTAAGAAGTGGTGACCATTAGGTTTTTG-3′

shLMNA-1: 5′-CCGGGCTGGAGCTAGAACAAACCAACTCGAGTTGGTTTGTTCTAGCTCCAGCTTTTTTG-3′

shLMNA-2: 5′-CCGGGAGTTGGTACTGGATCACATTCTCGAGAATGTGATCCAGTACCAACTCTTTTTG-3′

shIFFO2-1: 5′-CCGGAACGATAGGTCAGATCGAGCTCTCGAGAGCTCGATCTGACCTATCGTTTTTTTG-3′

shIFFO2-2: 5′-CCGGAATCCTTCTTCAAGACGAGAGCTCGAGCTCTCGTCTTGAAGAAGGATTTTTTTG-3′

### Immunoprecipitation

The cDNAs of distinct NHEJ genes (from hORFemone, v.8.1) were cloned onto pDonor, and mammalian expression plasmids were made by shuttling the genes to the destination plasmid pDEST26-Flag by LR cloning (Gateway Cloning, Invitrogen). For IFFO1 and IFFO2, the Flag-tag was changed into a maltose-binding protein (MBP)-tag. The expression plasmids were transfected into HEK293 cells [wild type (WT) or knockout] by PEI and were harvested 72 h later. The cell pellets were lysed with lysis buffer [25 mM Tris–HCl (pH 7.5),150 mM NaCl, 3 mM MgCl_2_, 5% glycerol, 0.5% NP-40, 1 mM dithiothreitol (DTT), 1 mM phenylmethylsulfonyl fluoride (PMSF), 150 U/ml benzonase nuclease, 5 µg/ml aprotinin, and 5 µg/ml leupeptin] on ice for 15 min. Then, the extract was ultracentrifuged at 439 800 × g for 15 min at 4°C. The supernatant was filtered and incubated with anti-FLAG M2 affinity gel or dextrin beads for 4 h, and the beads were washed with wash buffer [25 mM Tris–HCl (pH 7.5),150 mM NaCl, 3 mM MgCl_2_, 5% glycerol, 0.1% Tween-20, 1 mM DTT, and 1 mM PMSF]. The beads were eluted with 3× Flag peptides for 4 h or maltose for 20 min.

### Yeast two-hybrid assay

The *GAL4*-based Matchmaker yeast two-hybrid system (Clontech Laboratories, Mountain View, CA, USA) based on Gal4 was used for a yeast two-hybrid experiment according to the manufacturer’s instructions. The *IFFO2* gene was cloned into the Gal4 activation domain (AD) in the pGADT7 vector. The full-length gene, the truncated fragment of the 2B domain, and the two mutant Laminin A genes with E328K and R386M mutations were cloned into the Gal4 DNA-binding domain (BD) in the pGBKT7 vector. The corresponding vectors were co-transformed into Gold Yeast strains and were grown on a double-deficient Petri dish (without tryptophan and leucine) for 2–3 days and then expanded to a triple-deficient Petri dish (without tryptophan, leucine, and histidine) for 3–4 days. The yeast strain would grow on the triple-deficient Petri dish if two proteins interacted.

### Laser-induced foci

Cells were plated in a 35 mm glass-bottom culture dish (NEST Biotechnology) and cultured in DMEM (Invitrogen) containing 10% FCS in a 37°C incubator for 24 h. When the cell concentration reached ~60–80%, Lipo2000 (Mei5 Bio) transfection reagent was used to transfect green fluorescent protein (GFP)-tagged Lamin A, GFP-tagged IFFO2, or GFP-tagged IFFO1 with mCherry-tagged Lamin C, and the transfection time was between 24 and 48 h. After that, laser microirradiation was carried out with the MicroPoint Laser Illumination and Ablation System coupled to the Dragonfly Spin-disk system (ANDOR, with a wavelength of 365 nm) on a Leica DMi8 microscope with a ×63 plan apo oil immersion objective. Time-lapse images were acquired with ANDOR IQ3 software with an ANDOR IXON camera. Brifely, an image of the unperturbed cell was captured prior to laser microirradiation (time = 0 min). The microirradiation was performed at 65% input along an indicated line within the nuclei, with images acquired every 30 s for 10 min, and subsequently analyzed using ImageJ software. The mean fluorescence intensity of the irradiated region was compared with that of an unirradiated nuclear area, with background intensity subtracted from an independent region. The relative fluorescence intensity (RFI) of protein recruitment in the irradiated area was calculated using the following formula: RFI (*t*) = (*I_t_* – *I_b_*)/(*I_nu_* – *I_b_*), where *I_t_, I_b_*, and *I_nu_* represent the mean fluorescence intensities of the microirradiated area, the background, and the unirradiated nucleus, respectively.

### Generation of the DT40 knockout cells

DT40 knockout constructs for *IFFO2* were generated as previously reported [28] using the MultiSite Gateway Three-Flagment Vector Construction Kit (Invitrogen). The 5′ and 3′ arms were amplified from genomic DNA using the following primers: 5′ arm, GAGAACAACTTTGTATAGAAAAGTTGCCCAGCACCAAGTGCCTGCTTTG/GAGAACTGCTTTTTTGTACAAACTTGCTCCACGTGTTCGTTGGCAGGAG; and 

3′ arm, GAGAACAGCTTTCTTGTACAAAGTGGCGGTGTTTCCAGGCAGCGTTGTCAG/GAGAACAACTTTGTATAATAAAGTTGCTCGAGGAGACCCTTATGTCTTAGGGATG. They were cloned into pDONR P4-P1R and pDONR P2R-P3 vectors, respectively. The knockout constructs were generated by LR recombination of pDONR-5′arm, pDONR-3′arm, resistant gene cassette-containing pDONR-211, and pDEST R4-R3 destination vector. The knockout constructs were linearized before transfection. For *IFFO2* knockout constructs, the 5′ and 3′ arms were amplified using the primers: GAGAACAACTTTGTATAGAAAAGTTGGTCCATGTTGTGGTTCCAGC/GAGCTGGCTGCCTGCTCTCTGCTG and GAGAACAGCTTTCTTGTACAAAGTGGCAGCTGTTACATGTGGAAACAGCTA/GCACTGCCTCTCTTCTATCCGGTAC, respectively. The gene knockout clones were validated by genomic DNA PCR using primers for *IFFO2*: GTGGCTGCTCCTCCTTGATCCTCTG and GTCCCCAAATCCATTCTCCAGAGAG.

### Generation of IFFO2-knockout HT1080 and HEK293 cells

CRISPR/Cas9 [clustered regularly interspaced palindromic repeats (CRISPR)/CRISPR-associated protein 9] was used to generate IFFO2-deficient HEK293 and HT1080 cells. In brief, guide sequence (GGGCCGCAGCAACTCGGGCC or GAACCCAACCATTGACCTGC for human and mouse cells, respectively)-containing pX330 plasmids were transfected into cells [29]. Single colonies were harvested after 8–10 days of culture. The primers CTGGGCTCCAACATCCACCTCTTG/CCAGGGCCACGGCATTGGCGTTG and CTTTGCCTACCACCCATCTTT/CCTTGGCTTGAGTGCTTACCT for human and mouse cells, respectively, were used to amplify the *IFFO2* gene from the genome by PCR and the products were digested with ApaI or PstI. Colonies containing the expected fragments were then sequenced and examined by WB.

### Cell fractionation

Sus293 cells were harvested and washed once with phosphate-buffered saline (PBS; the pellet volume was estimated after PBS washing). Four volumes of CSK buffer (0.3 M sucrose, 20 mM HEPES, 0.5% Triton X-100, 50 mM NaCl, 3 mM MgCl_2_, 0.5 mM PMSF, 1 mM DTT, 5 μg/ml aprotinin, and 5 μg/ml leupeptin) was used to resuspend the cells and lysed on ice for 5 min. Then, the supernatant was annotated as “soluble” after centrifugation at 5000 × g for 3 min. The pellet was resuspended with four volumes of CSK buffer (containing an appropriate amount of DNase I) and digested at 100 rpm at 37°C for 1 h. After centrifugation at 5000 × g for 3 min, the supernatant was recorded as “DNase I” to remove chromatin components. The precipitate was digested on ice for 10 min with four volumes of 0.25 M (NH_4_)_2_SO_4_, and the supernatant was annotated as “0.25 M,” representing the tightly bound chromatin component. Similarly, the precipitate was digested with 0.65 M (NH_4_)_2_SO_4_, and the supernatant after centrifugation was recorded as “0.65 M,” representing the ultra-tight binding chromatin component. Finally, after washing with CSK buffer, the pellets were dissolved in four volumes of 8 M urea and labeled as “Matrix.”

### Colony formation assay

A sterile medium containing methylcellulose, DMEM-F12, and NaHCO_3_ was prepared before use. Different VP16 concentrations were added into the medium in a 6-well plate, and DT40 cells (WT and knockout) were evenly dropped. Cultivating in the 39°C incubator, the number of clones grown in each well was observed and counted after 6–14 days.

### DSB end mobility assay

HT1080 cells expressing mCherry–53BP1TD proteins were cultured in 35 mm glass dishes (NEST Biotechnology) at 37°C in a CO_2_-independent medium (Invitrogen) containing 10% FCS. For the rescue experiment, the cells were co-transfected with mCherry–53BP1TD and pCI–IFFO2 (WT/C473R) constructs. VP16 (2 μM) was used to treat the cells and induce DSBs at 37°C for 30 min. Subsequently, images were collected on the Delta Vision OMX SR super-resolution imaging system (GE Healthcare), equipped with a certified ×60 PLAPON objective lens, 1.42 NA. Before analysis, Max Intensity fast projection (softWoRx 7.0.0 software) was exploited to process the image through five cycles of deconvolution. The motion of DSB foci was analyzed by capturing images at intervals of 0.5 μm along the *Z*-axis across three sections, with a frequency of every 500 ms for 1 min. DSB motion was analyzed with the MATLAB tracking package “u-track” [30]. The mean square displacement (MSD) for each track as a time delay function was calculated. The scaling exponent (α) serves to characterize the mode of motion. In the context of subdiffusive motion associated with DSBs, a value of α < 1 is established as the criterion for filtering effective foci.

### CT assay

A CRISPR/Cas9 system was used to detect chromosomal translocations [27, 31, 32]. Brifely, a pair of CRISPR/Cas9 plasmids targeting two specific locations in the genome were transfected into the cells. To quantify the CT junctions generated by CRISPR/Cas9-induced DSBs, genomic DNA was purified from HEK293 WT, *IFFO1^−/−^, IFFO2^−/−^*, and *IFFO1^−/−^IFFO2^−/−^* cells. Then, the corresponding DNA segments were amplified with two pairs of primers. The PCR products were quantified by real-time PCR (qPCR), with expression levels normalized to that of the 53BP1 gene. For the Top3b–TNRC6C intra-translocation report system, the guide sequences were GGTCTCTCCTACCTCTAGAG and GAGTTCCAGAAGATGCCGCTT, respectively; and the primers used for qPCR were 5′-TGCTGTGCTGCGGAAGAGT-3′ and 5′-CAGCATTCCAACAGTGACATCAATG-3′. For the ERG–TMPRSS2 inter-translocation report system, the guide sequences were GTATTTGTTGCATGAGCTCC and GGGCTGACCTCCTAGGCCAT, respectively; and the primers used for qPCR were 5′-CTCAGGCCAGTGCACACCAGTTC-3′ and 5′-CTCCGTGCTGCTCCCTCTAGCA G-3′.

### Karyotype analysis

HEK293 and HT1080 cells infected with shRNA or transfected with plasmids were cultured in a 10 cm Petri dish. When the cell confluence reached 70%, the cells were irradiated with ionizing radiation at 1 Gy and recovered in a 37°C incubator for 3 h. Then 0.1 µg/ml colcemid was added to synchronize the cells for 2 h, and the metaphase cells were harvested. Subsequently, the collected cells were exposed for 15 min to a hypotonic solution (75 mM KCl) and fixed in 3:1 methanol/acetic acid. Then, metaphase spreads were prepared and stained with a 3% Giemsa staining solution. The prepared slides were observed with a ×100 objective lens of a Leica microscope, and the percentage of cells with cross-like quadrivalent chromosomes was counted in each sample.

### Random integration assay

Chicken DT40 cells were transfected with linearized pLox-puro plasmid. At 24 h post-transfection, 100 cells were plated into 96-well plates to evaluate the cell plating rate. Subsequently, 1 million cells were plated into 96-well plates with 0.5 μg/ml puromycin. After 5–7 days, the number of colonies was counted to determine the random integration rate, which was normalized by the cell plating rate.

### NHEJ/HR I-SceI reporter assays

Intracellular reporter assays were conducted following the previously described methods with slight modifications [33, 34]. Brifely, HEK293 cells were transfected with NHEJ reporter PimEJ5GFP or HR reporter pDRGFP, along with the pCBASceI plasmid to induce DNA DSBs. The RFP-N1 plasmid was co-transfected as a control to assess transfection efficiency. Cells were harvested 24 and 48 h post-transfection and subsequently analyzed by flow cytometry (CytoFLEX, Beckman, USA).

## Results

### IFFO2 interacts with XRCC4 and IFFO1

**Figure 1.**
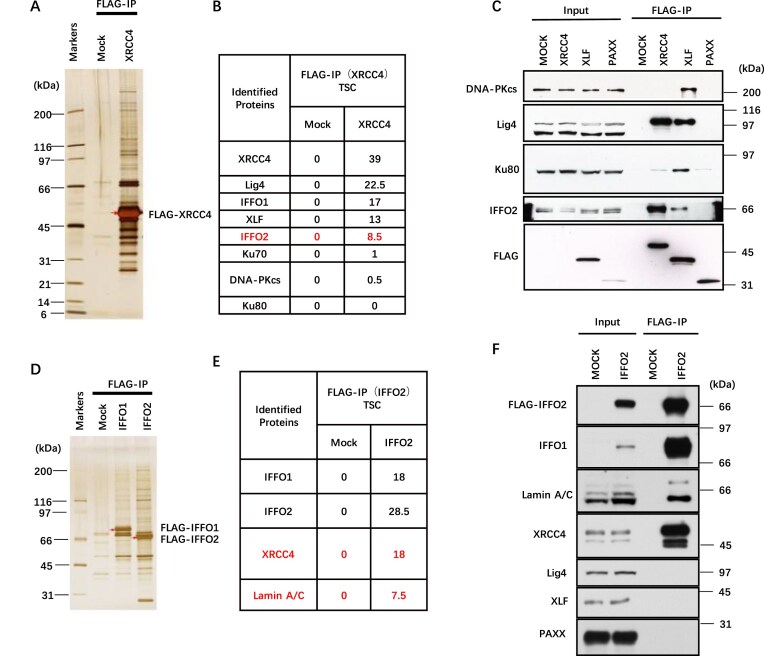
IFFO2 interacts with XRCC4 and IFFO1. (**A**) Silver staining of the eluted sample after XRCC4 IP. The red arrow points to the overexpressed XRCC4 protein. (**B**) The total spectral count (TSC) value of specific interacting proteins of XRCC4 was identified by MS (right). The number represents the average of two experiments. (**C**) Immunoblots showing the IPs of FLAG-XRCC4/XLF/PAXX in suspension 293 (sus293) cells. (**D**) Silver staining of the eluted sample after IFFO protein IP. The red arrow points to the overexpressed IFFO1 or IFFO2 protein. (**E**) The TSC value of specific interacting proteins of IFFO2 was identified by MS. (**F**) Specific NHEJ components were identified by WB following FLAG-IFFO2 IP.

An IP-MS-based interactome analysis was performed in our previous study [27]. In addition to IFFO1, its paralog IFFO2 was identified in XRCC4-associated complexes (Fig. 1A, B; Supplementary Fig. S1). The interaction of IFFO2 with XRCC4 was confirmed by WB (Fig. 1C). Conversely, only weak or no IFFO2 was detected in the immunoprecipitates of FLAG-XLF or FLAG-PAXX (Fig. 1C). Reciprocal IP followed by MS and WB revealed that XRCC4, rather than XLF or other NHEJ proteins, co-purified with FLAG-IFFO2 (Fig. 1D–F). Therefore, like IFFO1, IFFO2 is involved in the NHEJ process that mainly interacts with XRCC4. Additionally, IFFO1 co-purified with FLAG-IFFO2 (Fig. 1E, F), indicating that they link to each other.

### IFFO1 and IFFO2 form both homo- and hetero-oligomers through multiple interacting interfaces

**Figure 2.**
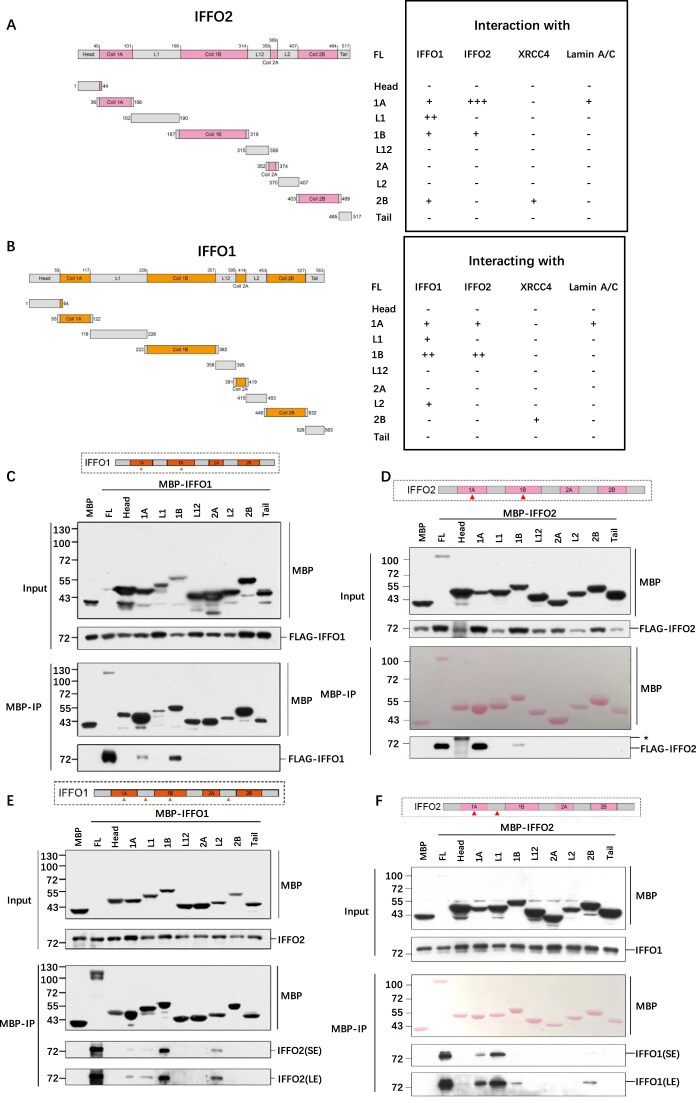
IFFO1 and IFFO2 form both homo- and hetero-oligomers through multiple interacting interfaces. (**A** and **B**) Left: IFFO2 and IFFO1 were divided into nine fragments according to structural characteristics, and the specific amino acid segments were marked. Right: summary of interacting partners of the indicated IFFO2 and IFFO1 fragments. Information on interactions is from (C–F). IP-WB results of MBP–IFFO1 (**C)** and MBP–IFFO2 (**D**) following the expression of FLAG-IFFO1 (or FLAG-IFFO2) in *IFFO1^−/−^IFFO2^−/−^* sus293 cells. IP-WB assessment of the fragments of MBP–IFFO1 (**E**) and MBP–IFFO2 (**F**) to interact with endogenous IFFO2 (or IFFO1) in *IFFO1^−/−^* (or *IFFO2^−/−^*) sus293 cells.

IFFO2 and IFFO1 have substantial sequence similarity in different species (Supplementary Fig. S1). They share the same functional domains, and they each consist of four coiled-coiled domains (1A, 1B, 2A, and 2B), three linkers (L1, L12, and L2), a head domain, and a tail domain (Fig. 2A, B). Among these domains, the four coiled-coiled domains show the highest similarity. Given these analogous features and their strong interaction shown in IP experiments, we assumed that they may interact to form homo-oligomers. To this end, we performed MBP pulldown assays in *IFFO1^−/−^IFFO2^−/−^* HEK293 cells (Supplementary Fig. S2A) to avoid interference from endogenous proteins. IFFO1s that were tagged with MBP and FLAG, respectively, were co-expressed, and FLAG-IFFO1 was enriched by MBP–IFFO1 (Fig. 2C). Similarly, the interaction of IFFO2 with itself was confirmed through co-IP of IFFO2 with FLAG and MBP tags (Fig. 2D). These results indicate that IFFO1 and IFFO2 interact with themselves and form homo-oligomers.

Furthermore, we constructed mutants with different truncations to identify the segments responsible for the homologous oligomerization of IFFO1 and IFFO2. Nine different truncated fragments of IFFO1 were labeled with MBP and expressed simultaneously with FLAG-labeled full-length IFFO1 in *IFFO1^−/−^IFFO2^−/−^* HEK293 cells (Fig. 2B). The 1A and 1B domains of IFFO1 are crucial for its dimerization, with the 1B domain having a stronger binding ability (Fig. 2C). Following the same strategy, FLAG-labeled IFFO2 full-length protein and different fragments of MBP-labeled IFFO2 were transfected into *IFFO1^−/−^IFFO2^−/−^* HEK293 cells. The results showed that the dimerization region of IFFO2 was the 1A and 1B domains, with the 1A domain having a stronger ability to bind to IFFO2 itself (Fig. 2D).

Next, we mapped the IFFO1 and IFFO2 domains responsible for hetero-oligomerization. Nine MBP–IFFO1 fragments were expressed in *IFFO1^−/−^* HEK293 cells, and the endogenous IFFO2 was detected by IP. Among different IFFO1 fragments, the 1B and L2 domains strongly interacted with endogenous IFFO2; the 1A and L1 domains also interacted, but to a lesser extent (Fig. 2E). Similarly, in different MBP–IFFO2 truncated proteins, the 1A and L1 domains interacted with endogenous IFFO1, whereas the interaction between the 1B and 2B domains and IFFO1 was weak (Fig. 2F). Together, IFFO1 and IFFO2 contain multiple interaction interfaces to form both homo- and hetero-oligomers.

### IFFO1 and IFFO2 have multiple interaction options, potentially forming complicated networks

To investigate the specific interactions between IFFO proteins in homo-oligomers, we constructed 1A and 1B domains of S protein, FLAG tag, and streptavidin-binding peptide (SFB)–IFFO1 (or SFB–IFFO2) and co-expressed them with either MBP–IFFO1 or MBP–IFFO2 1A or 1B in *IFFO1^−/−^IFFO2^−/−^* cells. For interactions among IFFO1 fragments, the level of SFB–1B pulled down by MBP–1B was the strongest, indicating that the same type of IFFO1 molecule mainly interacts through the 1B domain (Supplementary Fig. S2B). A weak interaction exists between 1A and 1B, suggesting that a cross-interaction is a secondary form, which is also consistent with the results of the pull-down of the full-length IFFO1, where 1A is a minor component (Fig. 2C). Similarly, the 1A and 1B domains of IFFO2 interacted with themselves or each other, with the strongest signal between the 1A domains (Supplementary Fig. S2C). These data indicate that both IFFO1 and IFFO2 can form homo-oligomers through multiple optional interaction modes.

We further examined the interactions among the fragments for hetero-oligomerization through the co-expression of the 1A and L1 domains of SFB–IFFO2 with either the 1A, L1, 1B, or L2 domains of MBP–FFO1. The 1A domain of SFB–IFFO2 was significantly pulled down by all four domains of MBP–IFFO1, with MBP–IFFO1_1B being the most effective (Supplementary Fig. S2D). Clearly, all four fragments of MBP–IFFO1 pulled down SFB–IFFO2_1A, although MBP–IFFO1 _1A failed to pull down SFB:IFFO2_L1 (Supplementary Fig. S2D). The interaction between MBP–IFFO1_1B and SFB–IFFO2_1A was most prominent (Supplementary Fig. S2D). These data suggest that IFFO1 and IFFO2 have multiple optional interaction modes for hetero-oligomerization. So, IFFO1 and IFFO2 may form a complicated network by rich interactions.

### IFFO2 bridges XRCC4 and the nucleoskeleton redundantly with IFFO1

**Figure 3.**
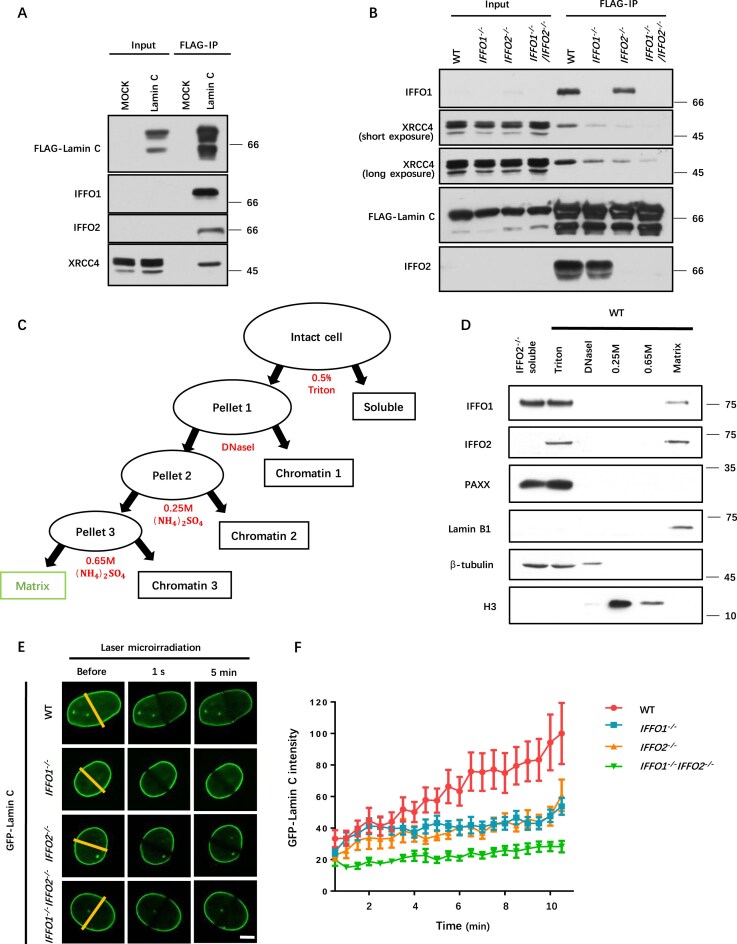
IFFO2 bridges XRCC4 and the nucleoskeleton redundantly with IFFO1. (**A**) Immunoblots showing the IPs of FLAG-tagged Lamin C. (**B**) The IPs of FLAG-Lamin C in WT, *IFFO1^−/−^, IFFO2^−/−^*, and *IFFO1^−/−^IFFO2^−/−^* HEK293 cells. (**C**) Experimental workflow of cell fractionation. (**D**) Immunoblot showing that IFFO2 is present in the nuclear matrix/skeleton of HEK293 cells. (**E**) Laser microirradiation-induced recruitment of GFP–Lamin C in WT, *IFFO1^−/−^, IFFO2^−/−^*, and *IFFO1^−/−^IFFO2^−/−^* HEK293 cells. After laser microradiation of the same intensity, imaging was performed and recorded for 5 min. Scale bar, 5 μm. (**F**) Quantitative statistical results of Lamin A laser-induced DSB assay after knocking out IFFO1 or (and) IFFO2. The relative GFP intensity was measured every 20 s after laser irradiation for 5 min over 40 cells per group.

In addition to XRCC4, IFFO1 and Lamin A/C (encoded by the *LMNA* gene) were also detected in the immunoprecipitates of FLAG-tagged IFFO2 (Fig. 1E, F). Reciprocal IP using FLAG-Lamin C revealed the presence of IFFO2 in the Lamin A/C-associated complexes (Fig. 3A). Using the same set of MBP–IFFO1/2 fragments, we found that the 1A domain of IFFO2 or IFFO1 interacts with Lamin A/C, while the 2B domain is required for XRCC4 binding (Supplementary Fig. S3A, B). Several mutations in Lamin A/C have been linked to various diseases [35, 36]. Among them, the E328K and R386M mutations located in the 2B coiled-coil domain of Lamin A eliminated the interaction between Lamin A and IFFO1, as demonstrated by yeast two-hybrid experiments [27]. Considering the similarity between IFFO2 and IFFO1, the same experiment was performed for IFFO2. Interestingly, both Lamin A mutants lost their interaction with IFFO2, and the 2B domain of Lamin A is sufficient and necessary for the interaction between Lamin A and IFFO2 (Supplementary Fig. S3C). Previously, we showed that the C519R mutation in the 2B domain of IFFO1 completely eliminated its interaction with XRCC4 [27]. Based on the conservation between IFFOs (Supplementary Fig. S1), we made the analogous mutant IFFO2-C473R. As expected, the interaction between XRCC4 and IFFO2 was no longer present for this mutant (Supplementary Fig. S3D). The interaction between IFFO1 and IFFO2 was further confirmed through IP utilizing endogenous antibodies (Supplementary Fig. S3E). These data indicate that IFFO2 interacts with XRCC4 in the same manner as with IFFO1.

Notably, XRCC4 is also present in the immunoprecipitate of FLAG-tagged Lamin C in HEK293 cells (Fig. 3A). This enrichment was dramatically decreased when IFFO1 or IFFO2 was absent, and it almost disappeared in *IFFO1^−/−^IFFO2^−/−^* cells (Fig. 3B), suggesting that IFFO1 and IFFO2 mediate the interaction between XRCC4 and Lamin A/C redundantly.

IFFO1, together with Lamin A/C, is present in the nucleoskeleton [27]. Considering the high similarity between IFFOs and the characteristics of intermediate filaments, we assumed that IFFO2 is also a component of the nucleoskeleton/nuclear matrix. We fractionated HEK293 cells (WT or *IFFO2^−/−^*) with high-concentration salt (Fig. 3C). Clearly, IFFO2 is present in the nuclear matrix like IFFO1 and Lamin B1 (Fig. 3D), supporting the idea that IFFO2 is part of the nucleoskeleton. Like IFFO1, it was also detected in the soluble fraction of the nucleus (Fig. 3D). Together, IFFO1 and IFFO2 act as scaffolds for XRCC4 and Lamin A/C, redundantly bridging the NHEJ machinery and nucleoskeleton.

### IFFO1 and IFFO2 recruit Lamin A/C to DNA damage sites in parallel

Since the interaction between XRCC4 and Lamin A/C depends on IFFO1 and IFFO2 (Fig. 3B), and the localization of Lamin A to DSB sites relies on IFFO1 [27], we reasoned that IFFO2 could also contribute to this process. The recruitment of GFP-tagged Lamin C to DNA damage sites was measured after laser microirradiation. The kinetics of the GFP signal in WT and distinct knockout cells (*IFFO1^−/−^, IFFO2^−/−^*, and *IFFO1^−/−^ IFFO2^−/−^*) were assessed (Fig. 3E). Like that in *IFFO1^−/−^*, the recruitment of Lamin A was seriously diminished in *IFFO2^−/−^* cells (Fig. 3E, F). Strikingly, the deletion of both IFFO1 and IFFO2 further decreased the recruitment of Lamin C (Fig. 3E, F), indicating that IFFO2 and IFFO1 redundantly recruit Lamin A to DSB loci.

### IFFO2 participates in NHEJ in parallel with XLF and IFFO1

**Figure 4.**
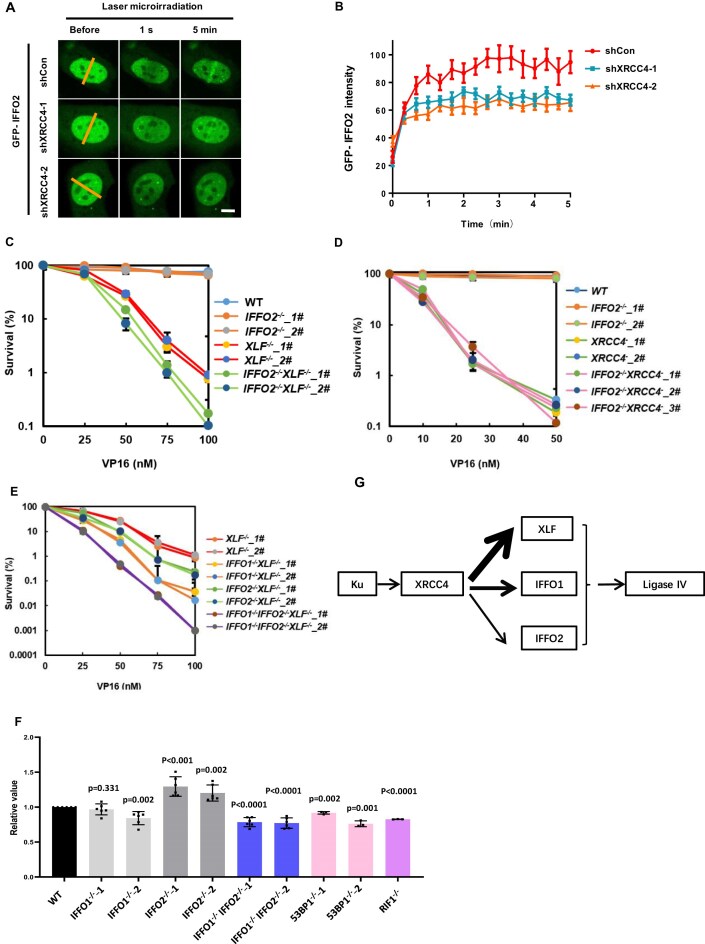
IFFO2 participates in the NHEJ pathway in parallel with XLF and IFFO1. (**A**) Live-cell imaging of GFP–IFFO2 at the sites of DNA damage after laser microradiation. The yellow lines indicate the positions of the laser treatment. Scale bar, 5 μm. (**B**) Quantitative statistics of GFP–IFFO2 accumulation after laser microradiation. (C–E) Colony formation assay of drug sensitivity to VP16 in (**C**) *IFFO2^−/−^, XLF^−/−^* and *IFFO2^−/−^XLF^−/−^*, in (**D**)* IFFO2^−/−^, XRCC4^−^* and *IFFO2^−/−^XRCC4^−^*, or in (**E**) *XLF^−/−^, IFFO1^−/−^XLF^−/−^, IFFO2^−/−^XLF^−/−^*, and *IFFO1^−/−^IFFO2^−/−^XLF^−/−^* cells. The average and standard deviation of the data in the figure are from three independent experiments. (**F**) The NHEJ efficiency of the indicated HEK293 cells was detected by fluorescence-activated cell sorting (FACS) after transfection of reporter plasmids for 24 h. The *P*-value was obtained by performing a two-tailed *t*-test comparing with the WT. Data are presented as the mean ± standard deviation (SD). Statistical significance was determined using a two-tailed Student’s *t*-test. Each dot represents the result of one independent experiment, with at least three independent replicates performed for each group. (**G**) Diagram of the relationship of the NHEJ ligase complex: ligase IV, XRCC4, XLF, IFFO1, and IFFO2. In the context of NHEJ, IFFO1 and IFFO2 are downstream of XRCC4 in a parallel way with XLF. Among these proteins, XLF plays a predominant role. Notably, in the absence of XLF, the contributions of IFFO1 and IFFO2 to the NHEJ process become more pronounced.

Due to the interaction between IFFO2 and XRCC4, we tested whether IFFO2 is directly involved in the NHEJ pathway and examined the recruitment of IFFO2 to DSB sites induced by laser microradiation in HT1080 cells. GFP–IFFO2 re-localized to DNA damage sites, especially near the lamina, like IFFO1 and Lamin C (Supplementary Fig. S4A). Notably, IFFO2 and Lamin C co-localized at damage sites on the equatorial plane and nuclear surface. Time-lapse monitoring showed that the concentrated GFP–IFFO2 signal was compromised upon XRCC4 deletion (Fig. 4A; Supplementary Fig. S4B), but not upon deletion of IFFO1, suggesting that XRCC4 is responsible for the recruitment of IFFO2 to DSB sites (Fig. 4A, B; Supplementary Fig. S4C, D).

The XRCC4-dependent recruitment of IFFO2 to DSB sites is consistent with the hypothesis that IFFO2 participates in the NHEJ process. DSBs caused by etoposide (VP16), a topoisomerase II inhibitor, are primarily repaired by NHEJ, manifested by the strong sensitivity in XRCC4-depleted DT40 cells (chicken lymphoma cells). We constructed *IFFO2^−/−^* DT40 cells and checked their sensitivity to VP16 (Supplementary Fig. S4E). Like *IFFO1^−/−^* cells, *IFFO2^−/−^* cells were not sensitive to VP16 (Fig. 4C, D).

We then examined the potential redundancy between IFFO2 and XRCC4 or XLF. IFFO2 was deleted in *XRCC4^−^* or *XLF^−/−^* DT40 cells. As illustrated, *IFFO2^−/−^XLF^−/−^* cells showed a stronger sensitivity to VP16 than *XLF^−/−^* cells (Fig. 4C), indicating that IFFO2 works in a subpathway different from XLF and plays a backup role in repairing VP16-induced DSBs. Notably, the deletion of IFFO2 did not enhance the sensitivity of *XRCC4^−^* cells to VP16 (Fig. 4D), suggesting that IFFO2 works in the same pathway as XRCC4 in NHEJ.

Since we concluded that IFFO1 plays a similar redundant role with XLF in NHEJ [27], we further analyzed the genetic relationship between IFFO1 and IFFO2 on the *XLF^−/−^* background and generated *IFFO1^−/−^IFFO2^−/−^XLF^−/−^* DT40 cells. Both *IFFO2^−/−^XLF^−/−^* and *IFFO1^−/−^XLF^−/−^* cells were more sensitive to VP16 than *XLF^−/−^* cells, and the phenotype of *IFFO1^−/−^XLF^−/−^* cells was more striking (Fig. 4E). Interestingly, the triple-knockout cells were more sensitive to VP16 than *IFFO2^−/−^XLF^−/−^* or *IFFO1^−/−^XLF^−/−^* cells, inferring that IFFO2 and IFFO1 participate in NHEJ as parallel backup subpathways in the absence of XLF (Fig. 4F). The NHEJ and HR reporter systems were tested to assess the repair efficiency [33, 34]. The NHEJ reporter PimEJ5–GFP and the HR reporter pDRGFP were transfected in HEK293 cells. Relative to WT cells, NHEJ repair efficiency in *IFFO1^−/−^* cells decreased to 97% and 84%, respectively, while in *IFFO1^−/−^IFFO2^−/−^* cells, NHEJ repair efficiency further declined to 79% and 77% after 24 h of transfection (Fig. 4F). Concurrently, the HR repair efficiency was also reduced in *IFFO1^−/−^IFFO2^−/−^* cells (Supplementary Fig. S4F). As controls, cells deficient in the core NHEJ factor 53BP1 exhibited repair efficiencies of 92% and 76%, whereas RIF1 knockout cells showed an efficiency of 83% at 24 h post-transfection (Fig. 4F). Furthermore, treatment with the DNA-PKcs inhibitor Nu7441 (5 μM) for 8 h resulted in a significant reduction of NHEJ repair efficiency to 48% (Supplementary Fig. S4G). At 48 h following transfection, the NHEJ repair efficiency in *IFFO1^−/−^IFFO2^−/−^* cells remained marginally lower than that of WT cells (95% versus 94%, respectively). In contrast, the HR repair efficiency did not exhibit a statistically significant difference (Supplementary Fig. S4H). These findings suggest that both IFFO1 and IFFO2 contribute to NHEJ repair, with their combined knockout exerting an effect on repair efficiency comparable with the loss of 53BP1 and RIF1. This indicates that IFFO1 and IFFO2 serve important auxiliary regulatory functions within the NHEJ repair pathway. These data are consistent with the finding that IFFO1 and IFFO2 bridge XRCC4 and Lamin A/C in parallel and recruit Lamin A/C to DSB sites.

XLF has two significant functions in NHEJ: stimulating the ligation activity of XRCC4–ligase IV and promoting the synapsis of broken ends [37]. Because IFFO1 and IFFO2 are absent in the ligase IV complex due to their incompatible interactions with XRCC4 (Fig. 1F) [27], they are likely to stimulate ligase IV activity redundantly with XLF. Therefore, we speculate that IFFO1 and IFFO2 may promote end synapsis redundantly with XLF in NHEJ-mediated DSB repair.

### IFFO2 immobilizes DSB ends with IFFO1

**Figure 5.**
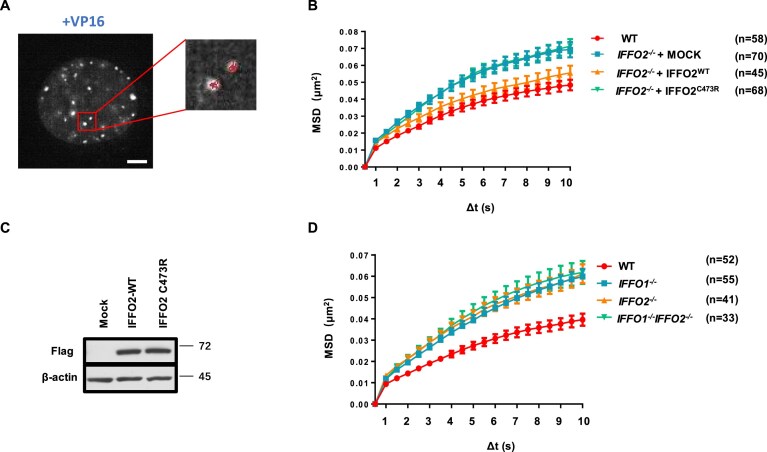
IFFO2 immobilizes DSB ends in the same pathway with IFFO1. (**A**) Live- cell imaging for the mobility measurement of DSB ends in HT1080 cells. Cells expressing mCherry–53BP1TD were treated with 2 μM VP16 for 30 min before imaging. Scale bars, 5 μm. (**B**) MSD of the mCherry–53BP1TD foci in *IFFO2^−/−^* cells transfected with WT or C473R mutant IFFO2. Means with standard errors of the mean (SEMs) of every time point are shown. (**C**) The WB results of FLAG-IFFO2 expression in HT1080 cells. (**D**) MSD value of the mCherry–53BP1TD foci in the *IFFO1^−/−^, IFFO2^−/−^*, and *IFFO1^−/−^IFFO2^−/−^* cells. Means with SEMs of every time point are shown.

Both Lamin A and IFFO1 have been shown to decrease the mobility of DSB ends [25, 27], so we tested whether IFFO2 has a similar role. As the Tudor domain of 53BP1 stably binds to DSB ends, we followed the movement of the mCherry-fused Tudor domain (53BP1TD, amino acids 1220–1711 ) by 3D time-lapse imaging in HT1080 cells (Fig. 5A) [25, 38]. After DSB induction by VP16, the broken ends in *IFFO2^−/−^* cells showed significantly higher mobility than that in WT cells (Fig. 5B). Strikingly, re-expression of WT IFFO2, but not its C473R mutant that lost the interaction with XRCC4, rescued the immobility of DSB ends in *IFFO2^−/−^* cells (Fig. 5B, C). These data suggest that IFFO2 anchors the broken ends through its interaction with XRCC4. Interestingly, the deletion of IFFO2 and IFFO1 did not further increase the mobility of DSB ends compared with single knockout cells (Fig. 5D), suggesting that IFFO1 and IFFO2 operate in the same pathway to immobilize broken ends, different from their genetic interaction in NHEJ-mediated DSB repair.

### IFFO2 prevents CTs in the same pathway as IFFO1

**Figure 6.**
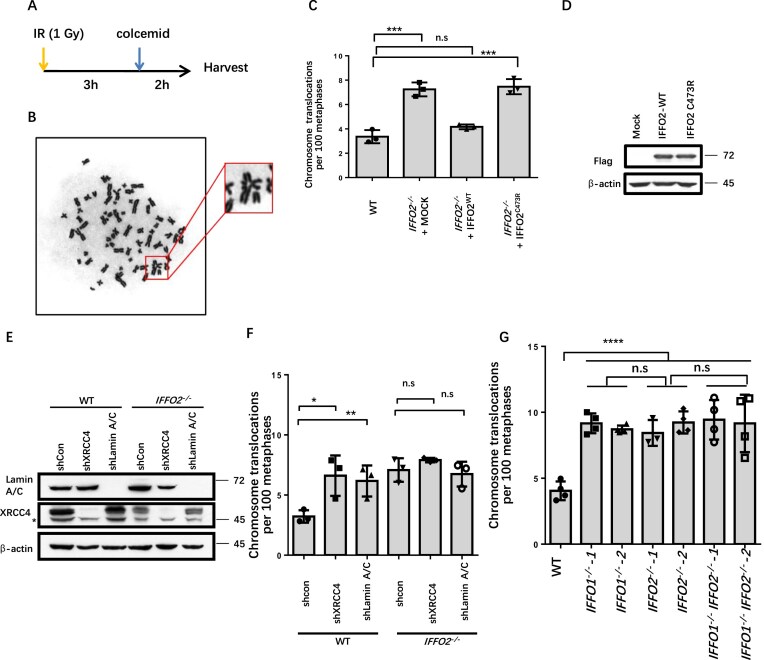
IFFO2 suppresses CTs. (**A**) Experimental workflow for karyotyping. HEK293 cells were treated with 1 Gy of ionizing irradiation and synchronized by colcemid to metaphase. (**B**) Schematic diagram of karyotype analysis of CTs. (**C**) The level of CT after transfecting IFFO2^−/−^ cells with WT or C473R mutant IFFO2. Means with SDs of three independent experiments are shown. ***P < 0.001; n.s, *P *> 0.05. (**D**) Measurement of the expression of FLAG-IFFO2 constructs in HEK293 cells using a western blot. (**E**) The protein level after knocking down XRCC4 or Lamin A/C in HEK293 WT or IFFO2 knocked-out cells. (**F**) The level of CT after infecting lentivirus shXRCC4 or shLMNA in HEK293 WT or *IFFO2^−/−^* cells. Means with SDs of three independent experiments are shown. *P < 0.05; **P < 0.01; n.s, *P *> 0.05. (**G**) Karyotype statistics for the number of cross-like quadrivalent chromosomes in HEK293 WT, *IFFO1^−/−^, IFFO2^−/−^*, and *IFFO1^−/−^ FFO2^−/−^* cells. Means with SDs of four independent experiments are shown. ****P < 0.0001; n.s, *P *> 0.05.

Considering that IFFO2 immobilizes broken DNA ends, we tested whether it inhibits CTs. We checked the rearrangement of chromosomes in response to severe DSBs by traditional karyotyping. HEK293 cells were synchronized to metaphase for karyotype observation after recovering from 1 Gy irradiation treatment for 3 h (Fig. 6A, B). Compared with WT cells, the frequency of CTs increased in *IFFO2^−/−^* cells (Fig. 6C). Also, supplementing WT IFFO2, but not IFFO2 with the C473R mutant, in *IFFO2^−/−^* cells rescued the CT frequency, indicating that IFFO2 suppresses CT through its interaction with XRCC4 (Fig. 6C, D). Consistently, depleting XRCC4 or Lamin A/C increased the level of CT in WT cells but not in *IFFO2^−/−^* cells (Fig. 6E, F). These results demonstrate that IFFO2 works in the same pathway as XRCC4 and Lamin A/C to inhibit CTs. Interestingly, *IFFO1^−/−^* cells showed a similarly elevated level of CTs to *IFFO2^−/−^* cells, while *IFFO1^−/−^IFFO2^−/−^* did not exacerbate CT compared with *IFFO1^−/−^* or *IFFO2^−/−^* cells (Fig. 6G), suggesting that IFFO2 and IFFO1 prevent CTs in the same pathway. Subsequently, we performed karyotype analysis in IFFO1 knockout, IFFO2 knockout, and double knockout cell clones within DT40 cells (Supplementary Fig. S5A), as well as HT1080 cells, and obtained consistent results (Supplementary Fig. S5B).

Additionally, we used CRSPR/Cas9-based reporter systems to measure CTs of site-specific DSBs (Supplementary Fig. S5C, D). As expected, the frequencies of both inter-CTs and intra-CTs were increased in *IFFO1^−/−^* and *IFFO2^−/−^* HEK293 cells, but they were not further increased in *IFFO1^−/−^IFFO2^−/−^* cells (Supplementary Fig. S5E). A similar result was also obtained in HT1080 cells (Supplementary Fig. S5F). Consistently, random integration was further decreased in *XLF^−/−^IFFO2^−/−^* cells compared with that in *XLF^−/−^* cells (Supplementary Fig. S5G). Together, these results reveal that IFFO2 and IFFO1 prevent CTs in the same pathway (Supplementary Fig. S6), consistent with their relationship in end immobilization but different from their genetic interaction in NHEJ.

## Discussion

The concept of the nucleoskeleton (or nuclear matrix) was first proposed in the 1970s [39]. In the process of DNA repair, nucleoskeleton proteins provide a structural framework to assist in the localization of repair complexes at the site of damage. The interactions and coordination between these nucleoskeleton proteins make the DNA repair network more complex but exquisite. For example, Lamin A/C and actin have dual roles in promoting c-NHEJ and HR, and Lamin B1- and actin–ARP2/3-dependent motion of chromatin favors HR [23, 40,–42]. SUN1 interacts with proteins in the process of c-NHEJ to inhibit NHEJ, promoting the HR process [23]. Our findings show that IFFO proteins play specific roles in c-NHEJ and enrich our understanding of the role of the nucleoskeleton in DNA repair.

Like IFFO1, IFFO2 was identified in XRCC4 interactors as a direct bridge between the nucleoskeleton and NHEJ. Genetic and cellular analyses revealed that IFFO proteins in the nucleoskeleton have two distinct functions in DSB processing. One is in NHEJ-mediated DSB repair. IFFO1 and IFFO2 show redundant functions in promoting end-joining only when XLF is absent (Supplementary Fig. S6A, B). This phenomenon is similar to that of PAXX [14]. We propose that IFFO1 and IFFO2 facilitate end synapsis, in which XLF plays a vital role, as does PAXX [14]. IFFO1 or IFFO2 alone be enough to form a “splint” for end synapsis through interaction with XRCC4 and Lamin A/C. Nonetheless, the possibility that both IFFO proteins and XLF are required for synapsis when the broken ends are “dirty” cannot be excluded, similar to the case of PAXX. The other function of IFFO proteins is DSB end anchoring to prevent CTs. This function is achieved by forming a polymeric network through IFFO proteins. IFFO2 and IFFO1 form homo- and hetero-polymers mainly through their N-termini, both of which use 1A and 2B domains to bind to Lamin A/C and XRCC4, respectively, ultimately fixing the DSB ends onto the nuclear scaffold (Supplementary Fig. S6B). A lack of IFFO proteins may prevent the formation of a stable nuclear lamina structure, leading to end drift that promotes CTs when multiple DSBs occur, ultimately promoting tumor development.

Here, by integrating previous experimental findings, a more comprehensive depiction of the interaction patterns of IFFO1 and IFFO2 is illustrated in Supplementary Fig. S6A (i). Both IFFO1 and IFFO2 homo-dimerize via their 1A and 1B coiled-coil domains. IFFO1 predominantly employs its 1B domain for self-interaction, while IFFO2 favors homotypic interaction through its 1A domain [Supplementary Fig. S6A (ii and iii)]. For both IFFOs, interactions between the 1A and 1B domains are of minor importance, and weak interactions occur for 1A–1A of IFFO1 or 1B–1B of IFFO2 [Supplementary Fig. S6A (ii and iii)]. Further studies on the interaction between different IFFO proteins have led to relatively complex situations, with multiple modes of interaction. (i) The interaction between the 1B domain of IFFO1 and the 1A domain of IFFO2 is the strongest, and IFFO1 (1B) can pull down the most endo-IFFO2, making it the primary interaction mode [Supplementary Fig. S6A (iv and v)]. In this mode, IFFO1 and IFFO2 interact in a forward parallel “head-to-tail” manner, where IFFO1 (L2) can also bind to IFFO2 (1A) and IFFO2 (L1), while mainly binding to IFFO2 (L1) [Supplementary Fig. S6A (iv and v)]. (ii) IFFO1 and IFFO2 also have a relatively strong forward parallel “head-to-head” interaction in the 1A–1A and L1–L1 regions, but there is spatial misalignment between the segments, which can explain the two weaker interactions between IFFO1 (L1)–IFFO2 (1A) and IFFO1 (1B)–IFFO2 (L1) [Supplementary Fig. S6A (vi and vii)]. These interaction modes indicate that the two IFFO proteins mainly interact through the N-terminus. (iii) There is also a significant interaction such as IFFO1 (L2)–IFFO2 (L1) or IFFO1 (L2)–IFFO2 (1A), with the former being stronger [Supplementary Fig. S6A (viii and ix)]. In addition to the forward parallel “head-to-tail” mode, there may also be a reverse parallel “head-to-tail” mode, where IFFO2 (1A) and IFFO2 (L1) bind to the IFFO1 (L2) region in a reverse parallel manner. This also reflects the intricate interweaving arrangement of nuclear lamina proteins in the nucleoskeleton rather than in a tidy arrangement with the same pattern. Different interaction modes may play different roles in various activities, which requires further research.

The nuclear context in which DSBs are situated plays a crucial role in determining the selection of repair mechanisms and the spatial dynamics of DSBs [43, 44]. Our findings from laser microirradiation experiments demonstrate that IFFO1 and IFFO2 exhibit accumulation at DSB sites located both centrally within the nucleus and adjacent to the lamina at the nuclear periphery (Fig. 4A; Supplementary Fig. S4A). Our findings indicate that the foci formed by IFFOs at the DSBs surrounding the nuclear periphery exhibit greater brightness in comparison with those located within the interior of the nucleus. This increased accumulation may be attributed to the pre-existence of the nuclear lamina, which may facilitate the recruitment or retention of free IFFOs at DSB sites. It is important to note that this does not imply an enhancement of NHEJ at the nuclear periphery. Under comparable conditions, the recruitment of core NHEJ factors, including DNA-PKcs, XRCC4, Ku70/80, ligase IV, XLF, and PAXX, at DSB sites located in the nuclear periphery does not exhibit a significant difference when compared with DSB sites within the nuclear interior [14, 45–50]. Previous research has observed decondensation of damaged heterochromatin domains, which may affect the choice of repair pathways [51, 52]. Heterochromatic DSBs undergo relocation to the periphery of heterochromatic domains [53], accompanied by enhanced mobility of the damage sites, and a higher likelihood of repair via HR [54–56]. Therefore, the brighter foci of IFFOs observed in the nuclear periphery may indicate their interaction properties with the nuclear lamina, rather than serving to influence the repair pathway choice within perinuclear heterochromatin regions or the spatial stability of damage sites. Research in yeast has demonstrated a continuous accumulation of DSBs at the nuclear periphery. There are two primary mechanisms for the repair of these DSBs: (i) the breaks are tethered to the nuclear pore via interactions with the Nup84 complex, utilizing either break-induced replication (BIR) or microhomology-mediated end joining (MMEJ) for their repair [57]; and (ii) the breaks are anchored to the inner nuclear membrane through interactions with Mps3, employing HR for the repair [58]. Although DSBs exhibit enhanced mobility during HR to facilitate the alignment of homologous sequences, their overall mobility within the nucleus remains constrained [59]. Cohesion complexes play a critical role in preventing the reconnection of distal DSBs during S phase, thereby contributing to genomic stability [60]. The localization of DSBs in mammalian cells remains consistent during the initial stages of repair [61]. Multiple DSBs on different chromosomes are repaired individually, without the presence of shared repair centers or movement towards the nuclear periphery [62]. DSBs that occur in the chromatin adjacent to the nuclear membrane are repaired within the nuclear lamina through NHEJ or a-EJ [63]. Consequently, the spatial proximity of DSBs is a critical determinant in the occurrence of chromosomal translocations in mammals. Under physiological conditions, a certain degree of DSB mobility facilitates the reconnection of broken ends. However, in the presence of extensive damage, the increased DSB mobility heightens the likelihood of incorrect reconnections [38]. DSB mobility is confined to a specific range in G_1_ phase to facilitate synapse formation and inhibit end separation [64]. In S/G_2_ phase, the presence of cohesion and base-pairing reduces the impact of DSB mobility on HR [38]. The real-time tracking of DSBs at the single-cell level demonstrated that DSBs away from the centromere induce a global mobility, display characteristics conducive to homologous search, and are critical for HR [65]. The findings of our study suggest that IFFO1/IFFO2 engages in interactions with XRCC4 and depends on XRCC4 for their localization at sites of damage. Consequently, it appears that the role of IFFO1/IFFO2 in the inhibition of DSB mobility is predominantly associated with the NHEJ process. During HR, the influence of IFFO1/IFFO2 on the mobility of ends may yield an opposing effect. This regulation may represent an evolutionary adaptation that differentiates between weak non-homologous interactions and robust homologous associations. However, the mechanisms underlying this process require further investigation.

For more than six decades, diagnostic methods based on the morphology, size, structure, and staining characteristics of cell nuclei have helped to accurately determine the type and staging of tumors, providing a basis for formulating treatment plans, and predicting patient prognosis [40]. Altered expression of nuclear lamins, particularly Lamin A/C, has been postulated to cause nuclear shape changes during cancer progression, although the underlying mechanisms remain unclear [40]. The spatial constraints of chromosomes, including the location of DNA breaks, the proximity of two erroneous break ends, and their mobility, significantly influence the results of CTs [66]. The deficiency of several critical NHEJ proteins, including MRN, DNA-PKcs, and ATM, facilitates CT by disrupting the maintenance of end synapsis [67, 68]. Despite exceptions observed in various experimental models, many studies on mice suggest that CT is inhibited by NHEJ [18, 66, 69, 70]. This inhibition is primarily attributed to the formation of synapsis and the spatial constraints of the proximity and mobility of these broken ends [66]. Our investigation indicates that IFFOs contribute to the suppression of CTs by enhancing NHEJ and stabilizing DSB ends. Specifically, DSB ends are recognized by NHEJ factors, including XRCC4, which forms a complex with IFFOs, thereby facilitating the synapsis of the ends and ensuring their proper connection. Furthermore, IFFOs, recruited by XRCC4, engage with Lamin A/C to construct the nucleoskeleton network that anchors the DSB ends. This NHEJ XRCC4–IFFO–Lamin A/C nucleoskeleton axis effectively stabilizes the DSB ends and mitigates the movement of DSBs [27]. Consequently, the deficiency of IFFOs leads to both compromised NHEJ and an abnormal Lamin A/C nucleoskeleton, which are significant factors contributing to CTs. In a previous study, we revealed that IFFO1–Lamin A/C expression negatively correlates with the frequency of gene fusion in multiple carcinoma types, consistent with an increasing range of tumors characterized by down-regulation of A-type lamin expression, including hematological malignancies [71, 72]. Similarly, in data from the NCBI, we found that the IFFO2 expression level in rhabdomyosarcoma patients with *PAX3* and *FOXO1* gene fusion, which is considered a predictor of adverse results in children with rhabdomyosarcoma [73], was lower than those in patients without *PAX3* and *FOXO1* gene fusion (Supplementary Fig. S7A). Similarly, CTs related to the mixed lineage leukemia (*MLL*) gene, found in 7–10% of acute lymphoblastic leukemia (ALL) patients, exhibited a lower IFFO2 expression level than those without MLL rearrangements (Supplementary Fig. S7B). Although these are individual cases, they show a promising potential for using dysregulated nucleoskeleton-related CTs in prognosis prediction of cancer patients.

## Supplementary Material

gkaf1354_Supplemental_Files

## Data Availability

The data underlying this article are available in the article and in its online supplementary material.
