# Supplementary material for: A nucleoskeleton network preserves genomic integrity by promoting NHEJ and restraining chromosome translocations
Source: Nucleic Acids Res. 2025 Dec 8;53(22):gkaf1354. doi: 10.1093/nar/gkaf1354 (PMC12684390; doi:10.1093/nar/gkaf1354)
Supplement: gkaf1354_Supplemental_Files [file gkaf1354_supplemental_files.zip › NAR-Supplementary material.pdf]

Supplementary material for

A nucleoskeleton network preserves genomic integrity by promoting NHEJ and restraining chromosome translocations

AUTHORS

Jingyan Liu[1, †], Xiuzhen Bai[1,2, †], Xinpeng Chen[1], Bohan Li[1], Huayu Zhao[1], Jiahui Wu[1], Yuanling Ye[3], Jiayi Yu[3], Zhenxin Yan[1], Rong Guo[1], Dongyi Xu[1, *], Wen Li [3, *]

[1] State Key Laboratory of Protein and Plant Gene Research, School of Life Sciences, Peking University, Beijing, China 100871.
[2] Biomedical Pioneering Innovation Center (BIOPIC), Peking University, Beijing, China 100871.
[3] Center for Medical Epigenetics, School of Basic Medical Sciences, Chongqing Medical University, Chongqing, China 400016.

* To whom correspondence should be addressed. Email: jcliwen@cqmu.edu.cn

Correspondence may also be addressed to Dongyi Xu. Email: xudongyi@pku.edu.cn

[†] The first four authors should be regarded as Joint First Authors.

Supplementary Figure 1

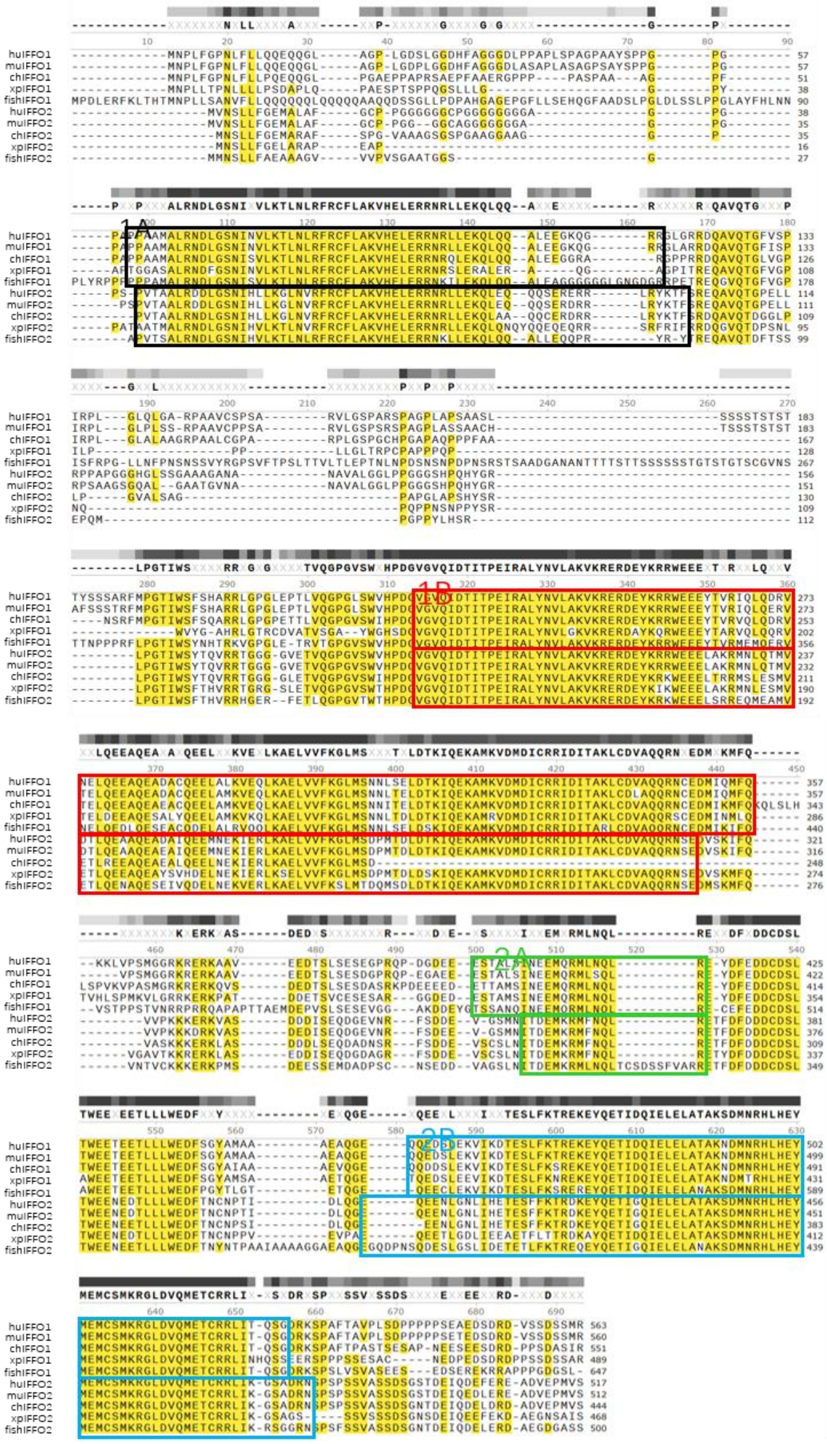

Supplementary Figure 1. Sequence alignment of IFFO2 homologs. Alignment results of IFFO2 and IFFO1 sharing homologous sequences in various species.

Supplementary Figure 2

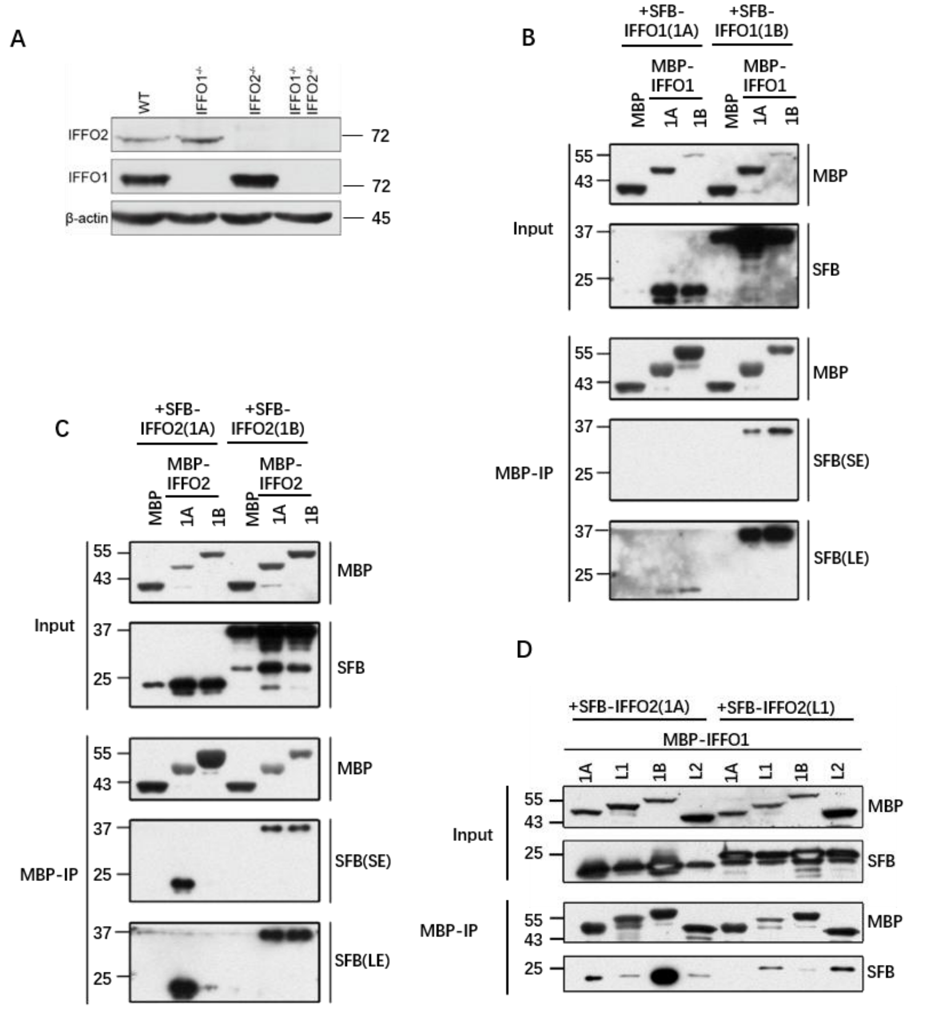

Supplementary Figure 2. IFFO1 and IFFO2 have multiple interaction options, potentially forming complicated networks. (A) Western blot verified the knock-out sus293. (B) MBP-IFFO1 fragments pulled down SFB-IFFO1 fragments to detect the specific interaction mode within the same IFFO proteins. (C) MPB-IFFO2 fragments pulled down SFB-IFFO2 fragments to detect the specific interaction mode within the same IFFO proteins. (D) MBP-IFFO1 fragments pulled down SFB-IFFO2 fragments to detect the specific interaction mode between two IFFO proteins.

Supplementary Figure 3

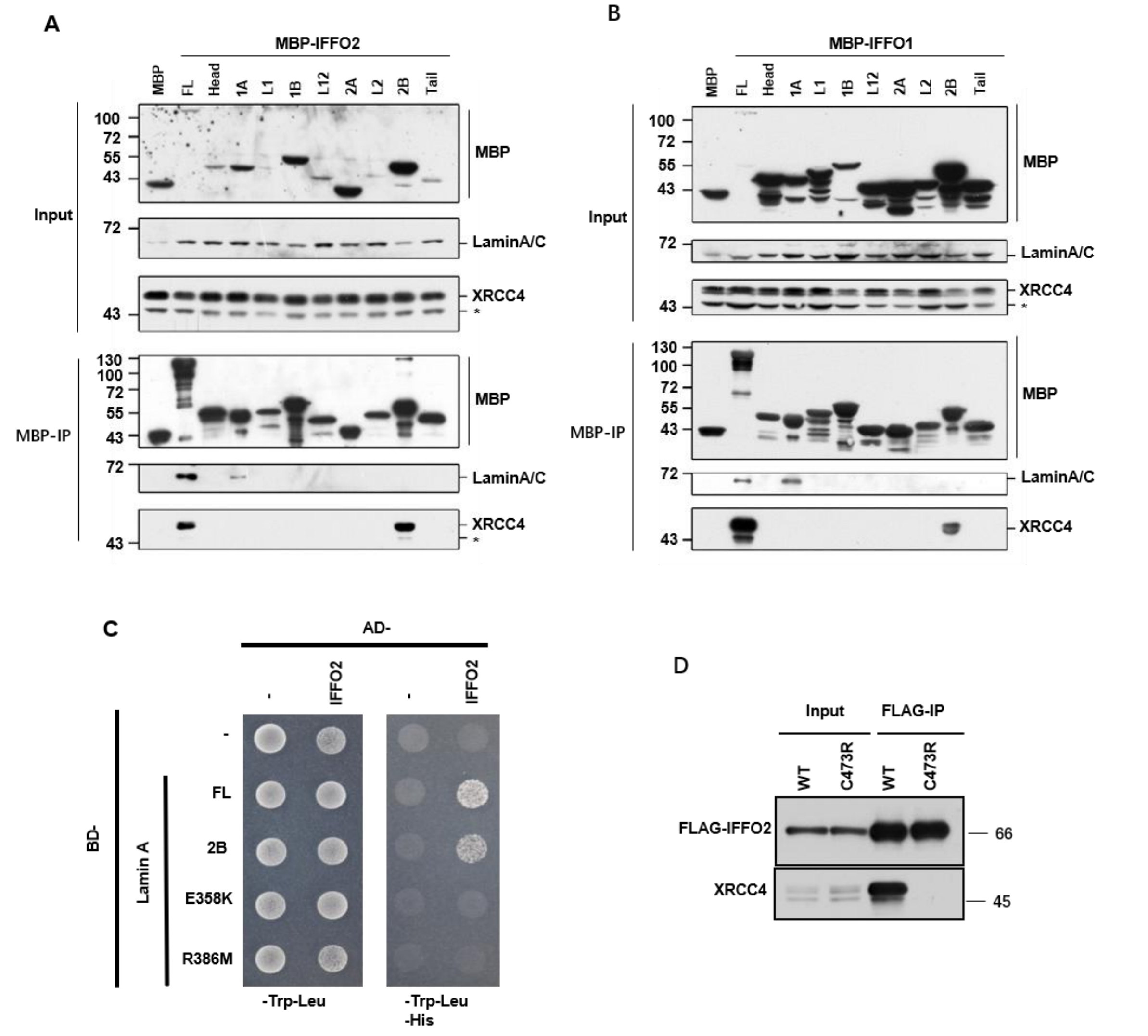

Supplementary Figure 3. Interconnection between IFFO2 and Lamin A/C or XRCC4. (A) IP-WB was used to explore the fragments of MBP-IFFO2 able to pull down Lamin A/C or XRCC4 in *IFFO1*$^{-/-}$*IFFO2*$^{-/-}$ cells. (B) IP-WB was used to assess the fragments of MBP-IFFO1 able to pull down Lamin A/C or XRCC4 in *IFFO1*$^{-/-}$*IFFO2*$^{-/-}$ cells. (C) A yeast two-hybrid experiment verified that IFFO2 interacts with Lamin A through its E358 and R386 sites on its 2B domain. (D) IP-WB verified that the C473R mutant of IFFO2 completely loses its interaction with XRCC4.

Supplementary Figure 4

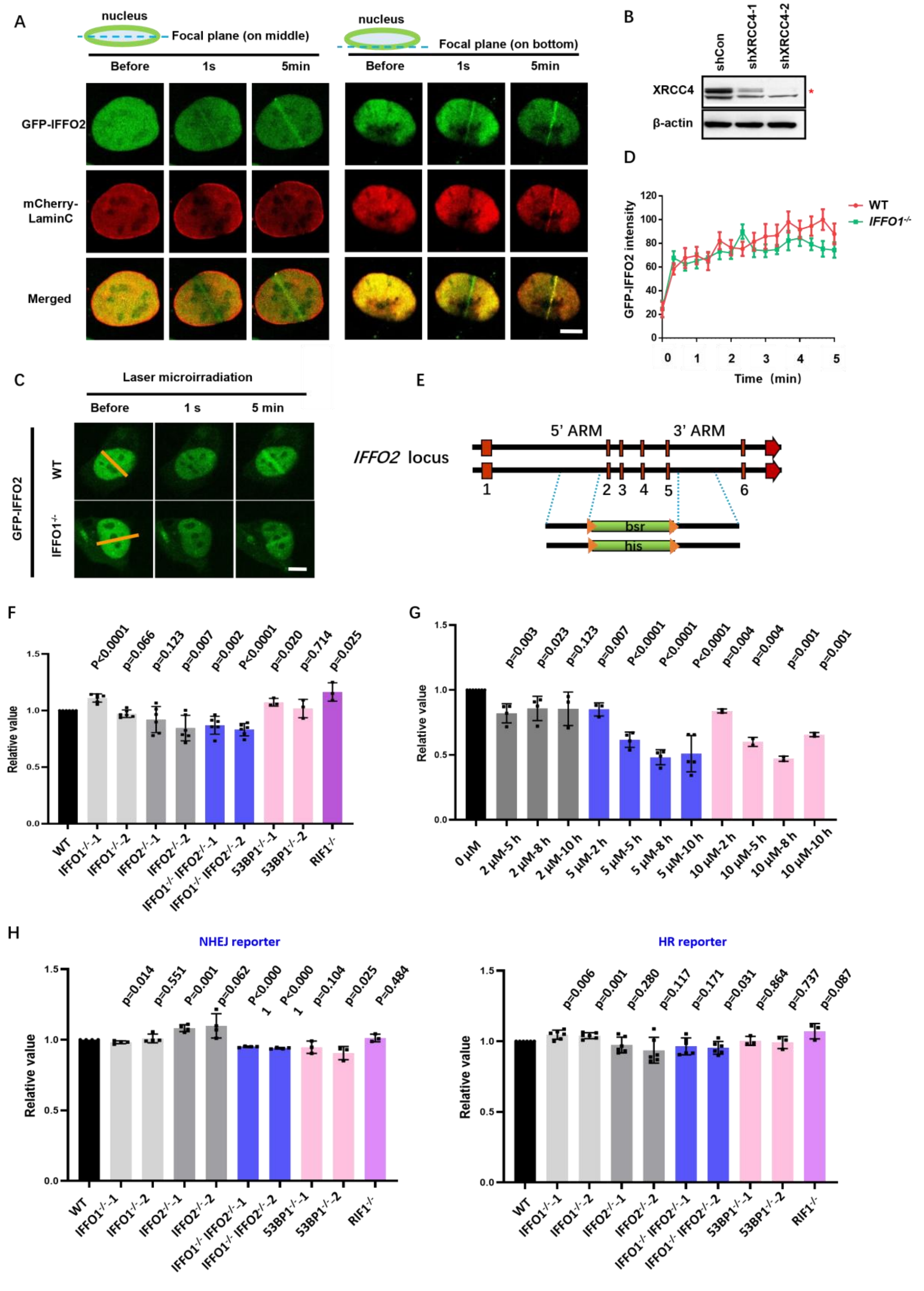

Supplementary Figure 4. IFFO2 participates in NHEJ repair. (A) Colocalization of GFP-IFFO2 and mCherry-Lamin C to the sites of DSB induced by laser micro-irradiation. The accumulated signals of both IFFO2 and Lamin C can be detected near the lamina and at pre-existing interior nucleoskeletal structures, respectively. (B) Western blot of XRCC4 knock-down efficiency. *, non-specific band. (C) GFP-IFFO2 was expressed in HT1080 cells (WT/ *IFFO1*$^{-/-}$). After laser micro-radiation of the same intensity, imaging was taken and recorded for 5 min. Scale bar, 5 µm. (D) Quantitative statistics of laser-induced IFFO2 foci in WT or *IFFO1*$^{-/-}$- HT1080 cells. The relative GFP-intensity was measured every 20 s after laser irradiation for 5 min over 30 cells per group. (E) IFFO2 knock-out DT40 construction. (F) The HR efficiency in the indicated cells after transfection of reporter plasmids for 24 hours. (G) HEK293 cells were treated with Nu7441 at the indicated concentrations and for the indicated durations, and NHEJ efficiency was analyzed by flow cytometry 24 hours after transfection. (H) The NHEJ (left) and HR (right) efficiency in the indicated cells after transfection of reporter plasmids for 48 hours. The p-value was obtained by performing a two-tailed t-test comparing with the WT. Data are presented as mean ± SD. Statistical significance was determined using a two-tailed Student's t-test. Each dot represents the result of one independent experiment, with at least three independent replicates performed for each group.

Supplementary Figure 5

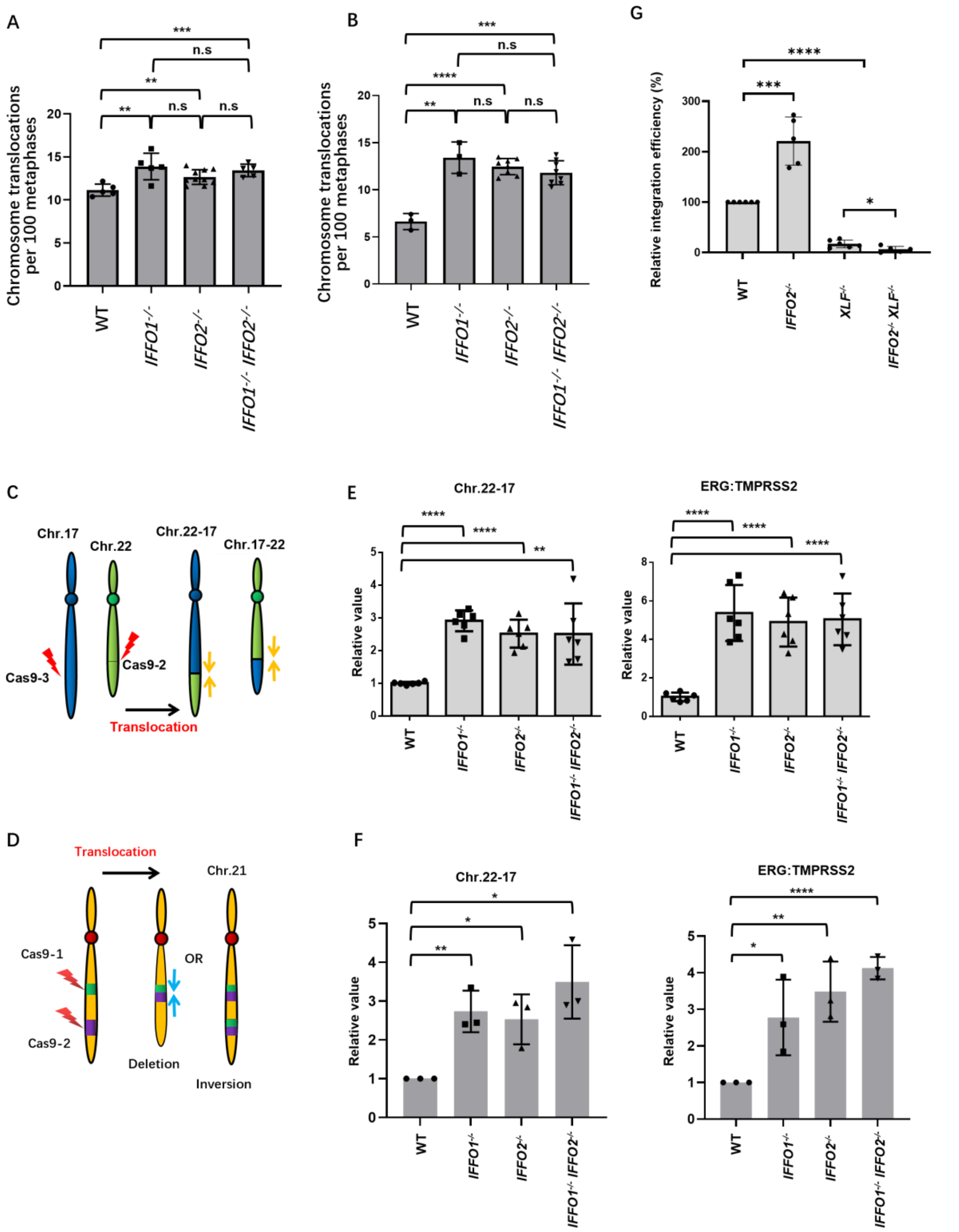

Supplementary Figure 5. IFFO2 immobilizes DSB ends to suppress CTs. (A) Karyotype statistics for the number of cross-like quadrivalent chromosomes in DT40 wild-type, *IFFO1*$^{-/-}$, *IFFO2*$^{-/-}$, and *IFFO1*$^{-/-}$ *IFFO2*$^{-/-}$ cells. Means with SDs of at least five independent experiments

are shown. **, P<0.01; ***, P<0.001; n.s, P>0.05. (B) Karyotype statistics for the number of cross-like quadrivalent chromosomes in HT1080 wild-type, *IFFO1*$^{-/-}$, *IFFO2*$^{-/-}$, and *IFFO1*$^{-/-}$ *IFFO2*$^{-/-}$ cells. Means with SDs of at least three independent experiments are shown. **, P<0.01; ***, P<0.001; ****, P<0.0001; n.s, P>0.05. (C) The red arrow indicates an example of an inter-CT. (D) The red arrow indicates an example of an intra-CT. (E) Inter-CT (Chr.22-17) and Intra-CT (ERG:TMPRSS2) in HEK293 cells. Means with SD of at least three replicates are shown. The error bar is the standard error, **, P<0.01; ****, P<0.0001. (F) Inter-CT (Chr.22-17) and Intra-CT (ERG:TMPRSS2) in HT1080 cells. Means with SD of at least three replicates are shown. The error bar is the standard error, *, P<0.05; **, P<0.01; ****, P<0.0001. (G) Random integration efficiency in DT40 cells. Means with SD of five replicates are shown. The error bar is the standard error, *, P<0.05 ; ***, P<0.001 ; ****, P<0.0001.

Supplementary Figure 6

A

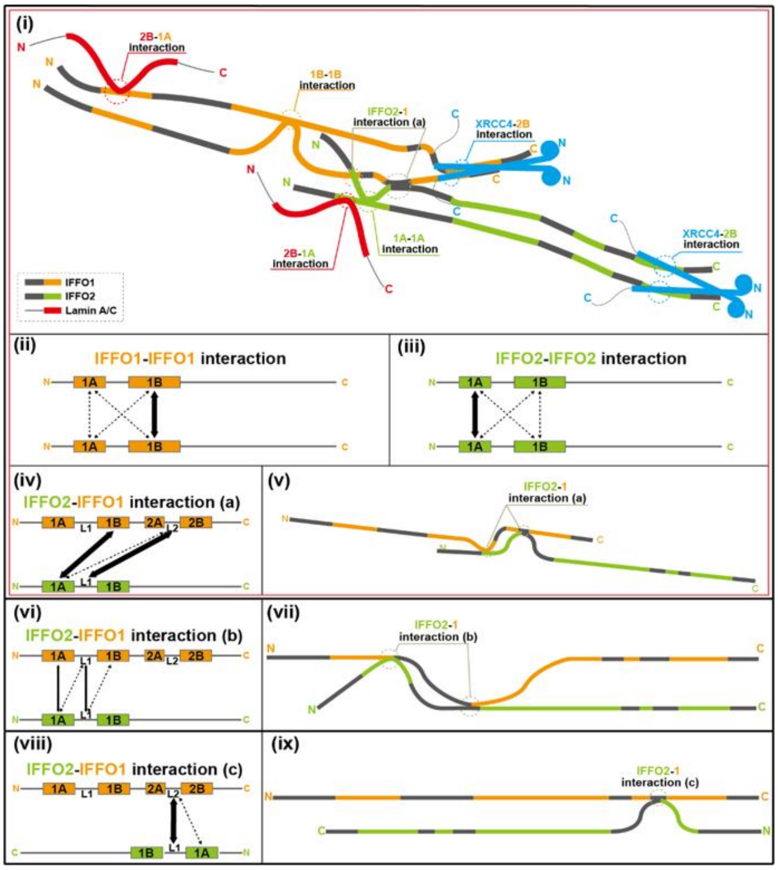

B

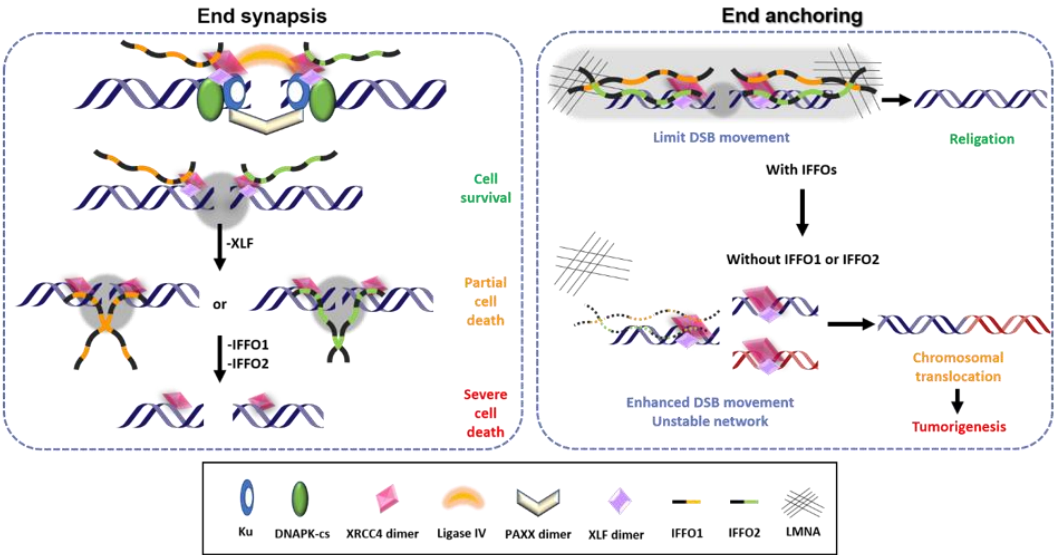

Supplementary Figure 6. Working model for how IFFO2 participates in NHEJ and suppresses CTs. (A) Model how IFFO1 and IFFO2 form a lamina network to bridge the nucleoskeleton and components of the NHEJ process. The interactions of IFFO proteins with each other are

diverse. (B) When DSB occurs, classic NHEJ-related proteins bind to the broken ends, and two steps occur. In end synapsis, soluble IFFO1 or IFFO2 redundantly participate in NHEJ repair and ligation of DSB as backups for XLF with lower effectiveness, giving rise to partial cell death. In end anchoring, IFFO family proteins form heteropolymers through their N-termini and connect with Lamins and the NHEJ protein XRCC4 through their 1A or 2B domains to stabilize the DSB ends. Loss of either IFFO protein may cause an unstable network, leading to the possibility that chromosomal translocation occurs when there is more than one DSB. This is one factor resulting in tumorigenesis.

Supplementary Figure 7

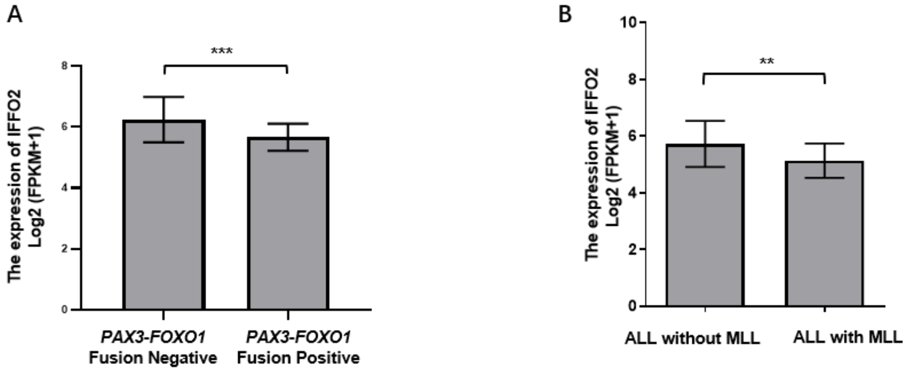

Supplementary Figure 7. IFFO2 expression level in cancer patients. (A) IFFO2 expression level in rhabdomyosarcoma patients with or without PAX3 and FOXO1 gene fusion. ***, $P<0.001$. (B) IFFO2 expression level in acute lymphoblastic leukemia (ALL) patients with or without MLL gene fusion. **, $P<0.01$.
