# Supplementary figures and images for: A nucleoskeleton network preserves genomic integrity by promoting NHEJ and restraining chromosome translocations
Source: Nucleic Acids Res. 2025 Dec 8;53(22):gkaf1354. doi: 10.1093/nar/gkaf1354 (PMC12684390; doi:10.1093/nar/gkaf1354)

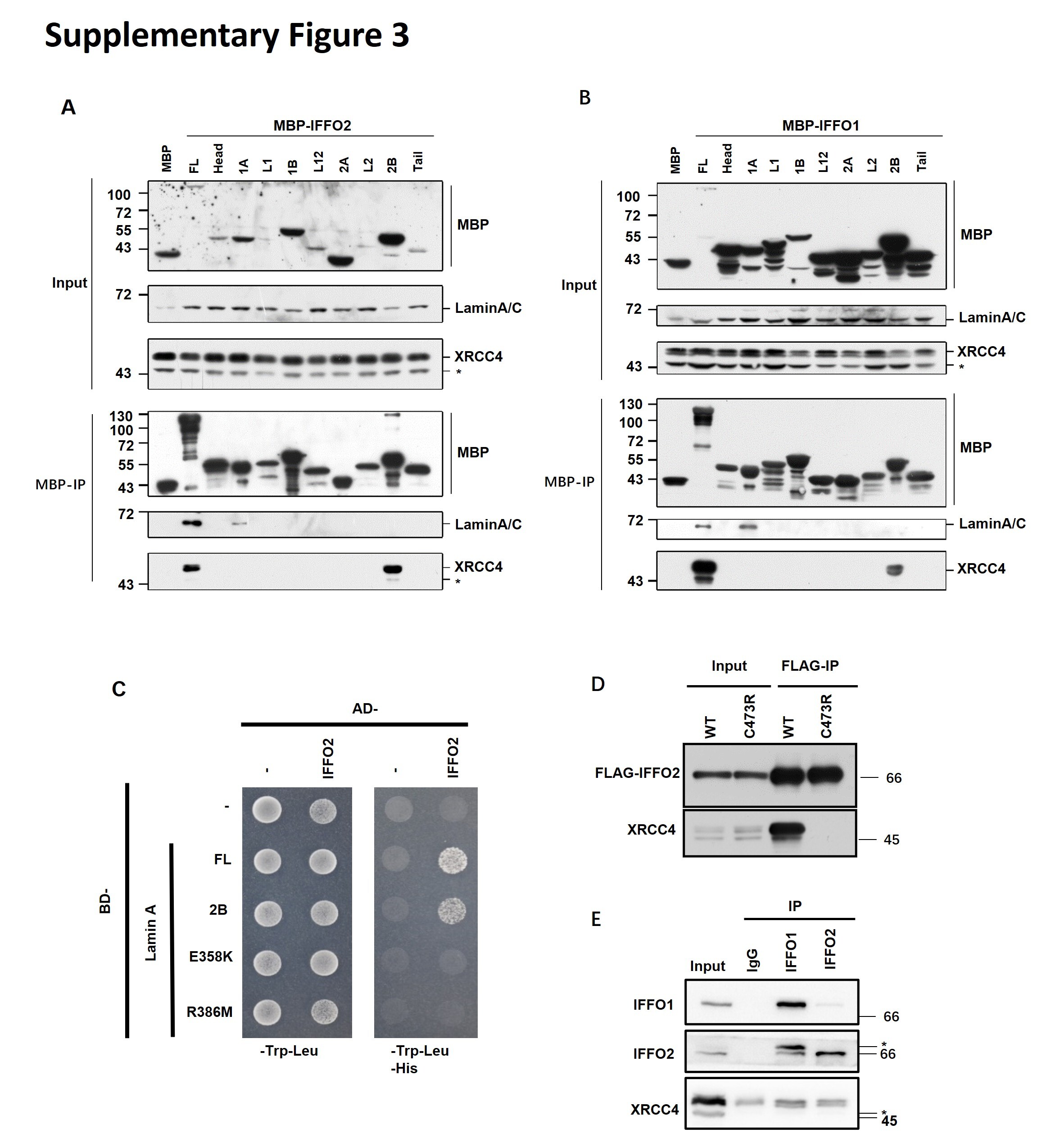

Supplement: gkaf1354_Supplemental_Files [file gkaf1354_supplemental_files.zip › Supplementary Figure 3.jpg]
